# Bio-guided isolation of potential anti-inflammatory constituents of some endophytes isolated from the leaves of ground cherry (*Physalis pruinosa* L.) *via ex-vivo* and *in-silico* studies

**DOI:** 10.1186/s12906-023-03934-9

**Published:** 2023-04-03

**Authors:** Asmaa Mahana, Hala M. Hammoda, Mona M. G. Saad, Mohamed M. Radwan, Mahmoud A. ElSohly, Doaa A. Ghareeb, Fathallah M. Harraz, Eman Shawky

**Affiliations:** 1 Department of Pharmacognosy, Faculty of Pharmacy, Alexandria University, Egypt, 21521 Alexandria Egypt; 2 Department of Pesticide Chemistry and Technology, Faculty of Agriculture, Alexandria University, 21545-El-Shatby, Alexandria, Egypt; 3 National Center for Natural Products Research, School of Pharmacy, University of Mississippi, MS 38677 Mississippi, USA; 4 Department of Pharmaceutics and Drug Delivery, University of Mississippi, MS 38677 Mississippi, USA; 5 Bio-screening and Preclinical Trial Lab, Biochemistry Department, Faculty of Science, Alexandria University, Alexandria, Egypt; 6 Biochemistry Department, Faculty of Science, Alexandria University, Alexandria, Egypt

**Keywords:** Endophytes, Alternaria, Physalis pruinosa, Anti-inflammatory, Ex vivo, Molecular docking

## Abstract

**Background:**

Due to the extensive potential of previously studied endophytes in addition to plants belonging to genus *Physalis* as a source of anti-inflammatory constituents, the present study aimed at isolation for the first time some endophytic fungi from the medicinal plant *Physalis pruinosa*.

**Methods:**

The endophytic fungi were isolated from the fresh leaves of *P. pruinosa* then purified and identified by both morphological and molecular methods. Comparative evaluation of the cytotoxic and ex vivo anti-inflammatory activity in addition to gene expression of the three pro-inflammatory indicators (TNF-*α*, IL-1*β* and INF-*γ*) was performed in WBCs treated with lipopolysaccharide (LPS) for the identified endophytes, isolated compounds and the standard anti-inflammatory drug (piroxicam). For prediction of the binding mode of the top-scoring constituents-targets complexes, the Schrödinger Maestro 11.8 package (LLC, New York, NY) was employed in the docking study.

**Results:**

A total of 50 endophytic fungal isolates were separated from *P. pruinosa* leaves. Selection of six representative isolates was performed for further bioactivity screening based on their morphological characters, which were then identified as *Stemphylium simmonsii* MN401378, *Stemphylium sp*. MT084051, *Alternaria infectoria* MT573465, *Alternaria alternata* MZ066724, *Alternaria alternata* MN615420 and *Fusarium equiseti* MK968015. It could be observed that *A. alternata* MN615420 extract was the most potent anti-inflammatory candidate with a significant downregulation of TNF-*α*. Moreover, six secondary metabolites, alternariol monomethyl ether (**1**), 3’-hydroxyalternariol monomethyl ether (**2**), alternariol (**3**), *α*-acetylorcinol (**4**), tenuazonic acid (**5**) and allo-tenuazonic acid (**6**) were isolated from the most potent candidate (*A. alternata* MN615420). Among the tested isolated compounds, 3’-hydroxyalternariol monomethyl ether showed the highest anti-inflammatory potential with the most considerable reductions in the level of INF-*γ* and IL-1*β*. Meanwhile, alternariol monomethyl ether was the most potent TNF-*α* inhibitor. The energy values for the protein (IL-1*β*, TNF-*α* and INF-*γ*)–ligand interaction for the best conformation of the isolated compounds were estimated using molecular docking analysis.

**Conclusions:**

The results obtained suggested alternariol derivatives may serve as naturally occurring potent anti-inflammatory candidates. This study opens new avenues for the design and development of innovative anti-inflammatory drugs that specifically target INF-*γ*, IL-1*β* and INF-*γ*.

**Supplementary Information:**

The online version contains supplementary material available at 10.1186/s12906-023-03934-9.

## Background

Natural sources hold the promise of discovering new structural classes with distinctive bioactivities for treatment of inflammatory diseases. *Physalis* is a tropical plant, which belongs to family Solanaceae [1]. *Physalis* plants are well known to be utilized in folk medicine for treating several ailments including malaria, hepatitis, asthma, rheumatism, diabetes and dermatitis also they were used as diuretic, antiseptic, antispasmodic, sedative and analgesic [2]. Recent medicinal reports showed that *physalis* plants exhibited hypoglycemic [3], anti-inflammatory [4], antitumor [5] and antioxidant effects [6]. There are more than 120 species of the genus *Physalis* in the world. *Physalis* is known locally in Egypt as Harankash, which is represented by three species: *P. ixocarpa* Brat, *P. pubescens* L and *P. pruinosa* L [7]. Meanwhile, the scarce reports on *P. pruinosa* L. prompted us to study this species. *P. pruinosa* was found to possess anti-inflammatory, hypoglycemic, antioxidant and antihyperlipidemic activities [3, 8].

Scientists are beginning to understand that plants may act as reservoirs for an infinite number of microorganisms, which are known as endophytes [9]. Endophytes are known to live in a symbiotic relationship interior plant tissue. Moreover, they are able to spend the most of their life cycle inside host plants. It is known that the secondary metabolites isolated from endophytes possess a broad spectrum of pharmacological activities, which include anticancer, antifungal, antiviral, anti-inflammatory and antibacterial activity [10]. For the past few decades, the discovery rate of active novel chemical entities has been clearly declined. Plant sources are being exhausted for new chemical entities discovery, which are utilized for various therapeutic purposes [9]. Thus, endophytic microorganisms were found to play an important role in the search for natural bioactive compounds. Only few plants have been studied to date for their endophytic variety and capacity to produce bioactive secondary metabolites. Endophytes are now recognized as exclusive suppliers of bioactive compounds [9]. It has been discovered that several endophytic fungal strains produce unique compounds with a beneficial effect as anti-inflammatory agents [11]. In recent studies, it is concluded that endophytic fungi are regarded as a valuable source for biologically active metabolites, which can be used to generate valuable novel anti-inflammatory analogs [12, 13].

Inflammation including acute and chronic ones can be considered as the body’s response to external stimuli like irritants, pathogens or infections. Chronic inflammation, however, can result from recurrent exposure to potentially hazardous substances or from inadequate control of acute inflammation [14]. Chronic inflammation-related disorders include cardiovascular, neurological and inflammatory bowel disease, which may develop as a result of persistent inflammatory reactions [15]. Additionally, chronic inflammation has a role in both physiological and pathological processes of a number of disorders, including Alzheimer’s disease, cancer, gout, obesity, depression and rheumatoid arthritis [16, 17]. Regulation of the inflammation process is performed *via* a precise modulation reaction between the cells and inflammatory mediators. The inflammatory mediators include cyclo-oxygenase and lipoxygenase enzymes, prostaglandin E2, nitric oxide, cytokines (tumor necrosis factor (TNF-*α*), interferons (IFN) and interleukins (ILs)) in addition to the transcription factor (NF-*Κ*B) [18]. They are released from the activated inflammatory cells (macrophages, neutrophils and eosinophils). The most widely used traditional treatments for inflammation are currently steroidal and non-steroidal anti-inflammatory drugs, which have analgesic, anti-inflammatory in addition to other curative effects. However, prolonged use of these medications may result in a variety of adverse reactions [19], including gastrointestinal harm (bleeding, gastric ulcers, etc.) [20], kidney and liver dysfunction [20]. However, rational use of the drugs recommends drugs with a higher level of safety. Hence, searching for natural good curative candidates without harmful side effects to replace the conventional anti-inflammatory medications is a pressing issue that must be resolved in the clinical management of inflammation-related diseases [21].

Due to the extensive potential of previously studied endophytes in addition to plants belonging to genus *Physalis* as source for anti-inflammatory candidates, the objective of the present study was to isolate for the first time some endophytic fungi from the medicinal plant *P. pruinosa*. Moreover, comparative evaluation of ex vivo anti-inflammatory activity of the isolated endophytic fungi on LPS-stimulated white blood cells was attempted followed by isolation of the main active metabolites from the most potent anti-inflammatory endophytic fungi. Assessment of ex vivo anti-inflammatory activity of the isolated compounds as well as the mechanisms underlying these effects using computer-aided molecular docking was performed.

## Methods

### Isolation of endophytic fungi

Healthy leaves of *P. pruinosa* L. (http://www.theplantlist.org/tpl1.1/record/kew-2549665) were collected from a farm owned by the University located in Moderyet El Tahrir District, Beheira Government, Egypt in April 2020. Plant authentication was done by Dr. Mahasen Sedky, Horticultural Research Institute, Cairo, Egypt. A voucher specimen (PP2020) was kept at the Department of Pharmacognosy, Faculty of Pharmacy, Alexandria University. Ethical approval for plant material collection was obtained from the research ethical committee at Faculty of Pharmacy, Alexandria University (PP2020/MN500). Plant collection complied with the IUCN Policy Statement on Research Involving Species at Risk of Extinction and the Convention on the Trade in Endangered Species of Wild Fauna and Flora. The collected fresh leaves were washed using tape water and then rinsed with distilled water. Furthermore, surface sterilization using 70% ethanol (1 min.) followed by 3.0% sodium hypochlorite (3 min.) was performed. Then rinsing the sterilized samples was done thrice using sterile distilled water (1 min. each). Every sterilized sample was cut into small pieces (approximately 5 mm) and then placed on petri dishes containing potato dextrose agar enriched with ampicillin (50 mg/L), which then incubated at 25 ± 2 °C. For culture purification, subculturing of the tips of the endophytic fungi, which grew out from the leaves was performed using other PDA plates [22, 23].

### Morphological characterization and molecular identification of endophytic isolates

Identification of the endophytic isolates was performed on the basis of macroscopic (fungus culture) and microscopic characters (spore shape and type) [24] in addition to molecular identification [25]. DNA extraction was performed as follow. The sample (200 µL) was mixed thoroughly with water (95 µL), solid tissue buffer (95 µL) and proteinase K (10 µL) in a microcentrifuge tube. Then the mixture was incubated at 55ºC for 2 h and centrifuged at 12,000 x g for 1 min. The aqueous supernatant was then transferred to a clean tube (300 µL) and mixed thoroughly with Genomic Binding Buffer (600 µL). In a collection tube, the mixture was transferred to a Zymo-Spin™ IIC-XL column followed by centrifugation at ≥ 12,000 x g for 1 min. In another collection tube, DNA Pre-Wash Buffer (400 µL) was loaded to the column followed by centrifugation at 12.000 xg for 1 min. Adding g-DNA wash buffer (700 µL) was performed followed by centrifugation at 12.000 xg for 1 min. Then 200 µL g-DNA wash buffer was added followed by centrifugation at 12.000 xg for 1 min. Finally, elution buffer (30 µL) was added and then incubated for 5 min. and centrifuged at 12.000 xg for 1 min. Polymerase Chain Reaction (PCR) was utilized for DNA amplification. The PCR was carried out using MyTaq Red Mix mastermix. ITS 1 (with base sequences TCCGTAGGTGAACCTGCGG) and ITS 4 (with base sequences TCCTCCGCTTATTGATATGC) (Invitrogen) were used as primers for DNA amplification. The PCR reaction mixture contained 25 µL MyTaq Red Mix polymerase, 8 µL DNA template, 1 µL forward primer (20 Pico mol), 1µL reverse primer (20 Pico mol) and 15 µL nuclease free water. The mixture was then applied to the thermal cycler (BioRad) using programmed PCR cycle. The amplified DNA was then submitted to a commercial service for sequencing and the base sequence was checked against freely accessible databases like GenBank using the Blast-Algorithmus.

### Cultivation and extraction of the fungal strain

For screening, isolation and identification of secondary metabolites from the cultivated fungi, mass growth was carried out using two methods. The first one [26] (solid rice medium cultivation) was used to cultivate *Stemphylium* [27] species which performed using 500 mL Erlenmeyer flasks containing 80 g rice and 120 mL distilled water (10 flasks), then incubated at 28 °C. Extraction of the fungal cultures was performed by sonication using methanol after three weeks of incubation. The methanolic extracts were then evaporated under reduced pressure. The second method [22] was used to cultivate *Alternaria* [27] and *Fusarium* [28] species, which was done by inoculation of the endophytic fungi in potato dextrose broth (PDB) medium (12 g potato dextrose per 500 mL distilled water in a 1 L Erlenmeyer flask (10 flasks for each strain) followed by incubation at 28 °C in a shaker incubator (120 rpm) for 2 weeks. The culture was then extracted using ethyl acetate and then sonicated at 40 ° C for 20 min. (three times). Collection of the ethyl acetate layer and then evaporation under vacuum was performed.

### Isolation of secondary metabolites from the endophytic fungi

The ethyl acetate extract of *A. alternata* MN615420 (800 mg) cultivated in PDB medium was chromatographed over a normal phase silica gel column (50 g, 2 cm X 90 cm) using hexane: ethyl acetate gradient elution with gradual increase in polarity (0-100%) to yield five fractions (Fractions A–E). Fraction A was purified by normal phase silica gel column chromatography (30 g, 1.5 cm X 80 cm) using a gradient solvent system of methylene chloride: methanol with gradual increase in polarity (10–80%) to afford compound **1** (15 mg), compound **2** (10 mg) and compound **3** (10 mg). Fraction C was subjected to normal phase silica gel column chromatography (30 g, 1.5 cm X 80 cm) using hexane: ethyl acetate gradient elution with gradual increase in polarity (20–90%) followed by sephadex LH-20 column eluted with methanol to obtain 5 mg compound **4**. Fraction D was separated by normal phase silica gel column chromatography (20 g, 1.5 cm X 60 cm) using a gradient solvent system of methylene chloride: methanol (20–90%) to yield an inseparable mixture of compounds **5** and **6**. Structure elucidation of the isolated compounds was done using NMR and MS techniques.

### Alternariol monomethyl ether (1)

Violet crystals, ESIMS negative m/z 271.1 [M – H]^−^. ^1^HNMR (500 MHz, DMSO): δ_H_ 2.75 (3H, s, 6’-CH_3_), 3.92 (3 H, s, 5-OCH_3_), 6.64 (1 H, d, *J* = 2.1 Hz, H-3’), 6.67 (1 H, d, *J* = 2.5 Hz, H-4), 6.74 (1 H, d, *J* = 2.1 Hz, H-5’), 7.25 (1 H, d, *J* = 2.5 Hz, H-6). ^13^CNMR (125 MHz, DMSO): δ_C_ 25.2 (6’-CH_3_), 56.2 (5-OCH_3_), 99.0 (C-2), 99.7 (C-3′), 102.0 (C-4), 103.8 (C-6), 109.1 (C-1’), 118.0 (C-5’), 138.2 (C-1), 138.7 (C-6’), 152.8 (C-2’), 159.0 (C-4’), 164.6 (C-3), 165.3 (C-5), 166.6 (C-7).

### 3’-Hydroxyalternariol monomethyl ether (2)

Yellow crystals, ESIMS negative m/z 287.2 [M – H]^−^.^1^HNMR (500 MHz, DMSO): δ_H_ 2.68 (3H, s, 6-CH_3_), 3.79 (3H, s, 5-OCH_3_), 6.76 (1H, d, *J* = 2.0 Hz, H-4), 6.99 (1H, s, H-5), 7.28 (1H, d, *J* = 2.0 Hz, H-6), 12.51 (1H, s, 3-OH). ^13^CNMR (125 MHz, DMSO): δ_C_ 25.1 (6-CH_3_), 56.5 (5-OCH_3_), 100.1 (C-2), 100.3 (C-4), 104.8 (C-6), 111.4 (C-1), 117.5 (C-5), 128.5 (C-6), 131.5 (C-3), 138.4 (C-1), 142.2 (C-2’), 146.5 (C-4), 165.0 (C-3), 165.2 (C-7), 167.1 (C-5).

### Alternariol (3)

Colorless crystals, ESIMS negative m/z 257.0 [M – H]^−^. ^1^HNMR (500 MHz, DMSO): δ_H_ 2.71 (3H, s, 6’-CH_3_), 6.38 (1 H, d, *J* = 2.2 *Hz*, H-3’), 6.65 (1 H, d, *J* = 2.0 Hz, H-4), 6.72 (1 H, d, *J* = 2.2 Hz, H-5’), 7.26 (1 H, d, *J* = 2.0 Hz, H-6), 11.70 (1 H, s, 3-OH). ^13^CNMR (125 MHz, DMSO): δ_C_ 25.7 (6’-CH_3_), 97.8 (C-2), 101.3 (C-4), 102.1 (C-3′), 104.8 (C-6), 109.4 (C-1′), 118.0 (C-5′), 138.5 (C-1), 138.8 (C-6’), 153.1 (C-2′), 158.8 (C-4′), 164.5 (C-3), 165.1 (C-7), 165.9 (C-5).

### *A*-Acetylorcinol (4)

Yellow crystals, ESIMS positive m/z 167.3 [M + H]^+^. ^1^HNMR (500 MHz, DMSO): δ_H_ 2.07 (3 H, s, H-9), 3.45 (2 H, s, H-7), 6.04 (2 H, m, H-2, H-6), 6.09 (1 H, m, H-4). ^13^CNMR (125 MHz, DMSO): δ_C_ 29.6 (C-9), 50.5 (C-7), 101.2 (C-4), 108.2 (C-2, C-6), 136.9 (C-1), 158.8 (C-3, C-5), 206.6 (C-8).

### Mixture of tenuazonic acid (5) and allo-tenuazonic acid (6)

Viscous, yellow oil, ESIMS negative m/z 196.1 [M – H]^−^.^1^HNMR (500 MHz, CD_3_OD): δ_H_ 0.81(m, H-10), 0.82 (m, H-10), 1.16 (m, H-11), 1.35–1.42 (m, H-9), 1.90 (m, H-8), 2.35 (s, H-7), 3.61 (s, H-5), 3.78 (s, H-5). ^13^CNMR (125 MHz, CD_3_OD): δ_C_ 11.6 (C-10), 15.2 (C-11), 22.8 (C-9), 25.6 (C-7), 36.4 (C-8), 36.8 (C-8), 63.7 (C-5), 65.5 (C-5), 102.5 (C-3), 177.5 (C-2), 177.8 (C-2), 193.6 (C-6), 193.7 (C-6).

### Evaluation of cytotoxicity and anti-inflammatory activity of the tested extracts and isolated compounds

#### Human white blood cells isolation and cultivation

Collection of a whole blood specimen was performed using a sterile heparin tube, then an aliquot (1 mL) of blood was transferred into a centrifuge tube (15 mL), which filled to capacity with fresh cold lysing solution. The tube was then inverted at room temperature for ~ 10 min. till the liquid turned into clear red. Centrifugation of specimens was done at 2000 rpm (4 ^o^C) for 10 min., then decantation of supernatant was performed and the tubes were permitted to drain. In 10 mL cold phosphate buffer saline (137 mM NaCl, 10 mM Na_2_HPO_4_, 2.7 mM KCl and 10 mM KH_2_PO_4_, pH 7.4), the pellets (WBCs) were suspended Followed by recentrifugation. The pellets were then resuspended using RPMI culture medium, which contained 2% L-glutamine and 10% fetal bovine. Then, dye exclusion method for assessment of WBCs viability and counting was utilized. Mixing of 50 µL cell suspension with the same volume of trypan blue staining solution (0.5%) was performed followed by hemocytometer loading. In each of the four corner quadrants (A, B, C, D), viable and nonviable cells were counted [29].

### Calculations

**N / ml = Mean of WBCs counting x10** ^4^ **× D**

N: Number of viable or nonviable cells.

D: Sample dilution (1:1 with the trypan blue).

$$\% \,Cell\,viability = \frac{{Number\,of\,viable\,\,cells}}{{Total\,number\,of\,cells}} \times 100$$

To use the cells for assays, at least 90% of them must be viable after incubating the culture in CO_2_ incubator for six days. Seeding of WBCs was performed (100,000 cells/ well) in a 96 well cell culture plate followed by incubation in CO_2_ incubator (5% CO_2_ and 90% relative humidity at 37 °C).

All experiments were performed in accordance with relevant guidelines and regulations.

#### Evaluation of cytotoxicity of the studied extracts and isolated compounds relative to piroxicam (MTT assay)

200 µL of cultured medium (containing 100,000 WBCs / well (96-well cell culture plate)) were seeded with variable concentrations of the studied extracts/ isolated compounds (0, 3.125, 6.25, 12.5, 25 and 50 µg/mL) in RPMI medium without both fetal bovine serum and piroxicam (the standard anti-inflammatory drug) then incubation was performed in CO_2_ incubator for 72 h (37 °C, 5% CO_2_ and 90% relative humidity). Then MTT solution was added to each well after 72 h of incubation. 20 µL volume of plates were incubated in CO_2_ incubator for 3 h to enable the MTT to be reacted. Following incubation, the plates were centrifuged for 10 min. at 1650 rpm, then the medium was discarded. MTT byproduct, formazan crystals, was re-suspended using DMSO (100 µL) and then measurement of readings was performed using optima spectrophotometer at wavelength 570 nm for safe dose determination that cause 100% cell viability [30].

### The % viability was calculated as follow: (AT-Ab /AC-Ab) x 100

**AT** = Mean absorbances of cells treated with different concentration of each studied extract or isolated compound.

**AC** = Mean absorbances of the control untreated cells (only culture medium).

**Ab** = Mean absorbances of cells treated with vehicle of the studied extracts or isolated compounds (RPMI without fetal bovine serum).

Using the percent viability derived from each studied extract/ isolated compound serial dilutions, the cytotoxicity assay of the drug was expressed as EC100 that calculated using the Graphpad Instat software (GraphPad Software Inc, California).

#### Determination of the effective anti-inflammatory concentrations (EAICs) of the used treatments in lipopolysaccharides (LPS)-stimulated human WBC’s culture

50 µL culture medium, which contained 100,000 of human WBCs, was dispensed per well in a 96 well plate. Inflammation induction was performed by adding 50 µL of LPS (20 µg/mL) to the plated cells followed by incubation in CO_2_ incubator. After 24 h, centrifugation of the plate (1650 rpm) was done for 5 min., afterward the supernatants were discarded. After that, 200 µL of crude extracts, isolated compounds or piroxicam were added using serial concentrations (0, 3.125, 6.25, 12.5, 25 and 50 µg/ml in culture media). Only cell culture medium was present in the control cells. Plates incubation occurred for extra 72 h in CO_2_ incubator. Following incubation, cell proliferations were assessed using MTT (as previously mentioned). Cell proliferations assessment was done using stimulation index (SI) [31].

**Stimulation index** = (mean absorbance of LPS-stimulated cells or LPS-stimulated cells treated with different concentrations of the studied extracts or isolated compounds / absorbance of control untreated cells).

Instate graph pad was used to determine the effective anti-inflammatory concentrations (EAICs) of the studied extracts and isolated compounds that could restore the aberrant proliferation of LPS-stimulated cells to the normal proliferation of control, untreated cells (SI = 1).

#### Extraction of RNA of untreated and treated LPS-stimulated human white blood cells

Suspension of cell pellets was then made in solution R1 (50 µL), then it was mixed thoroughly for 30 s and incubated at room temperature (1 min.). 300 µL solution R2 was added, mixed thoroughly for 30 s and then centrifuged for 3–5 min. at 4ºC. In a spin column, the supernatant was transferred followed by centrifugation for 30 s at 14,000 rpm (4ºC). After removing the flow-through, working wash buffer (300 µL) was added into the spin column followed by centrifugation for 30 s (repeated twice). Recentrifugation of the spin column was performed at 10,000 rpm (1 min.) and then transferred to a sterile micro centrifuge tube (1.5 mL). Elution buffer (30 µL) was added to the central of the membrane, then incubated at room temperature (1 min.) and then centrifuged for 30 s at 14,000 rpm at 4ºC. Determination of the optical density (OD) of the extracted RNA was performed through measuring the absorbance (A260 nm) and purity (A280 nm) using spectrophotometer then kept in -80 °C till real time PCR [32].

#### Synthesis of cDNA from RNA extracted from untreated and treated LPS-stimulated human white blood cells

Total RNA or nuclease-free water (2 µg) in addition to oligo dT primer (1 µL) were mixed gently with nuclease-free water in PCR tubes in a total volume of 12 µL, centrifuged, incubated at 65ºC (5 min.) using PCR machine and then put back on ice instantly. 5X reaction buffer (4 µL), dNTPs mix (2 µL), RNase inhibitor (1 µL) and reverse transcriptase (1 µL) or nuclease-free water (1 µL) in place of reverse transcriptase for reverse transcriptase negative control were mixed gently with prior mixture, spin down and then incubated at 42ºC (60 min.) followed by heat inactivation at 70ºC (5 min.) in PCR machine [32].

#### Determination of IL-1β, TNF-α, INF-γ and GADPH expression level by real time polymerase chain reaction (PCR)

In PCR tubes, a volume of 12.5 µL of 2 X SYBR green master mix was mixed with 5µL of cDNA 0.5 µL of 10 pico moles/mL forward primer and 0.5 µL of 10 pico moles/mL reverse primer for each primer. As for the reference tube, 0.5 µL of 10 pico moles/mL forward primer of *β*-actin and 0.5 µL of 10 picomoles/mL for reverse primer of *β*-actin were added. The other tube was used as a non-template control (NTC), to assess for reagent contamination or primer dimers by adding 1 µL of nuclease-free water instead of template used. The tubes were gently mixed with 6.5 µL nuclease free water without creating bubbles (bubbles will interfere with the fluorescence detection) and spinned for few seconds. Samples were placed in the cycler and start the program as follows; 1 cycle of 95ºC for 10 min. (initial denaturation), followed by 40 cycles of 95ºC for 15 s (denaturation), 60ºC for 30 s (annealing) and 72ºC for 30 s (extension) [32].

### Calculations

**Expressions fold levels of gene calculated by:**

**ΔCt** normal = Ct normal untreated cells – Ct reference.

**ΔCt** tested sample = Ct tested sample-treated cells – Ct reference.

**ΔCt** induced =- Ct LPS-exposed cells – Ct reference.

**In case of genes:**

**ΔΔCT** tested sample = ΔCt tested sample– ΔCt normal.

**ΔΔCT** induced = ΔCt induced – ΔCt normal.

**In case of GAPDH:**

ΔΔCT_tested plant extract_ = ΔCt _normal_ – ΔCt _tested plant extract_.

ΔΔCT_induced_ = ΔCt _normal_ – ΔCt _induced_.

**Fold change in gene expression = log (2** ^−ΔΔCT^ **)**

**Where**:

**Ct** tested sample: Threshold cycle value of genes of extracted mRNA of the studied extracts/isolated compounds treated-LPS-stimulated WBCs which is the cycle number at which fluorescence produced during a reaction crosses the fluorescence threshold.

**Ct** reference: Threshold cycle value of GAPDH which is utilized in normalization.

**Ct** normal: Threshold cycle value of genes of extracted mRNA of untreated control WBCs.

**Ct** induced: Threshold cycle value of gene of extracted mRNA of LPS-stimulated WBCs.

### Primers

**Table:** 

TNF-α	F, CTCTTCTGCCTGCTGCACTTTG
	R, ATGGGCTACAGGCTTGTCACTC
IL-1β	F, CCACAGACCTTCCAGGAGAATG
	R, GTGCAGTTCAGTGATCGTACAGG
INF-γ	F, GAGTGTGGAGACCATCAAGGAAG
	R, TGCTTTGCGTTGGACATTCAAGTC
GAPDH	F, GGATTTGGTCGTATTGGG
	R, GGAAGATGGTGATGGGATT

### In silico docking studies

The Schrödinger Maestro 11.8 package (LLC, New York, NY) was employed in order to predict the binding mode of the most potent bioactive compounds [33].

#### Ligand structures preparation

The two-dimensional structures of the isolated compounds along with the reference drug; piroxicam (CID 54,676,228) were obtained from PubChem database (https://pubchem.ncbi.nlm.nih.gov/) of the National Centre for Biotechnology Information in sdf file format. ChemDraw software (Cambridge So Corporation, Cambridge, USA) was used to draw the structures that weren’t included in PubChem database after searching through various literature. The chemical structure of each substance was imported into the Maestro 11.8 panel interface (Maestro, version 11.8, 2018, Schrodinger, USA) once the dataset was generated. The 3D structure was created using the LigPrep 2.3 module (LigPrep, version 2.3, 2018, Schrodinger, USA), which was also used to search for various conformers. To geometrically optimize each ligand structure and compute partial atomic charges, the OPLS (OPLS 2005, Schrodinger, USA) force field was used.

#### Target protein preparation

The crystal structures of human IL-1*β* (PDB ID 4DEP) [34], TNF-*α* (PDB ID 2AZ5) [35] and INF-*γ* (PDB ID 6E3K) [36] were obtained from the RCSB Protein Data Bank (PDB). Using the protein preparation module of Schrodinger’s Maestro Molecular Modelling Suit (Schrödinger Release 2015-2), the protein crystallographic structure was loaded and created.

#### Grid box generation

A grid box with dimensions of 15 × 15 × 15 ^o^A around the centroid of the co-crystallized ligand and a grid spacing of approximately 0.375 ^o^A was used to define the active site for docking on the prepared X-ray crystal structure of the targeted proteins. The targeted proteins crystal structure was subjected to binding interaction examination using this grid box size with the isolated compounds along with the reference drug.

#### Molecular docking

The Glide 11.8 module (Glide, version 11.8, 2018, Schodinger, USA) was used to perform flexible docking on the minimized and refined compounds while utilizing the Glide default parameters. The Glide-Dock program’s empirical scoring functions was used to generate modeling scores. In order to investigate the most favorable binding modes of ligands, the Maestro interface, which displayed the 2D and 3D ligand-target protein interactions (including hydrogen bonds, ion-pair and hydrophobic interactions) was employed.

#### Docking process validation

A pose selection approach was used to re-dock piroxicam ligand into the various binding sites of 4DEP, 2AZ5 and 6E3K in order to successfully validate the docking protocol. This was followed by estimating the root mean square deviation (RMSD) of the anticipated pose to the co-crystallized one piroxicam. The results showed high docking accuracy because they were less than 1 ^o^A (a preselected cut-off/threshold value). The re-docked complexes were superimposed onto the reference co-crystallized complexes with low root mean square deviation (RMSD) values (0.52 Å for 2AZ5, 0.59 for 6E3K and 0.65 for 4DEP).

### Statistical analysis

Each experiment was carried out in triplicate. All biological data were statistically analyzed using one-way analysis of variance (ANOVA), with p values less than 0.05 being regarded as significant. A neighbour-joining phylogenetic trees were constructed using MEGA X. Graphpad Instat (GraphPad Software Inc, California) was used to calculate EC100 values and construct the dose response curves.

## Results and discussion

### Identification of the isolated endophytic fungi

**Fig. 1:**
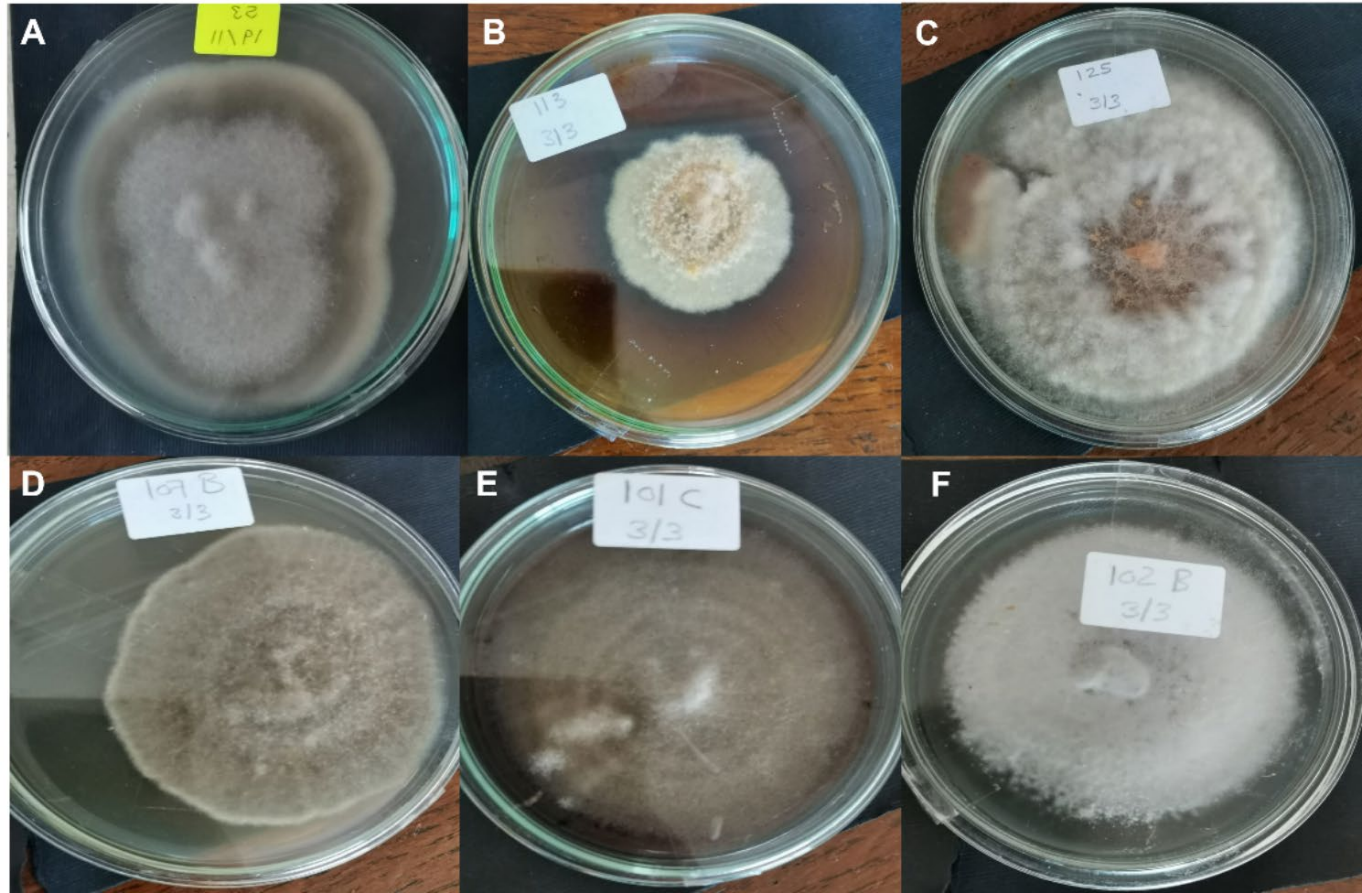
The endophytic fungi colonies on PDA medium after 7 days incubation. (**A**) *Alternaria alternata* MN615420, (**B**) *Stemphylium sp*. MT084051, (**C**) *Fusarium equiseti* MK968015, (**D**) *Alternaria alternata* MZ066724, (**E**) *Stemphylium simmonsii* MN401378, (**F**) *Alternaria infectoria* MT573465

**Fig. 2:**
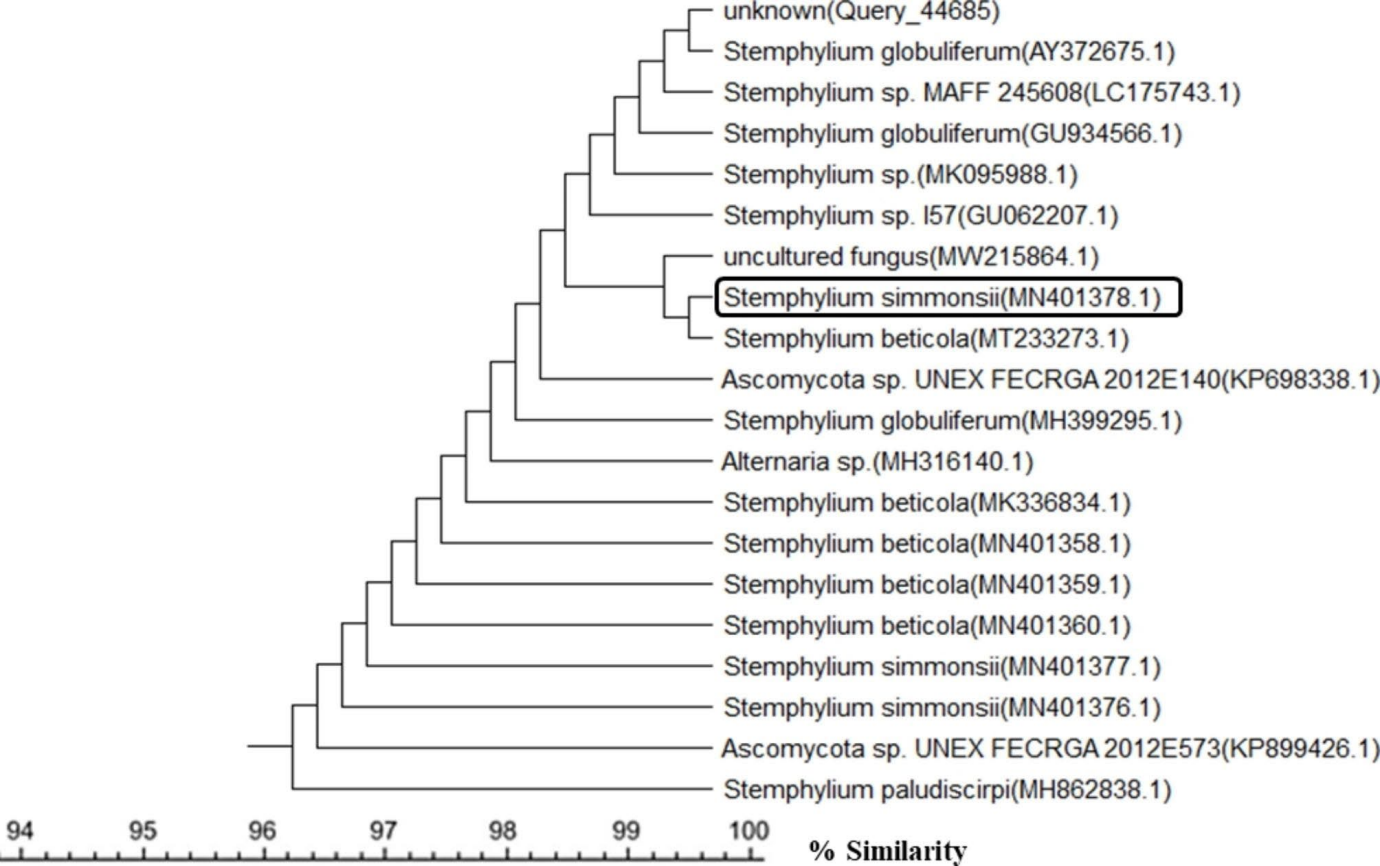
Phylogenetic tree based on neighbour-joining method using MEGA X of the identified endophytic fungus (*Stemphylium simmonsii* MN401378) isolated from *P. pruinosa* leaves and related sequences

**Fig. 3:**
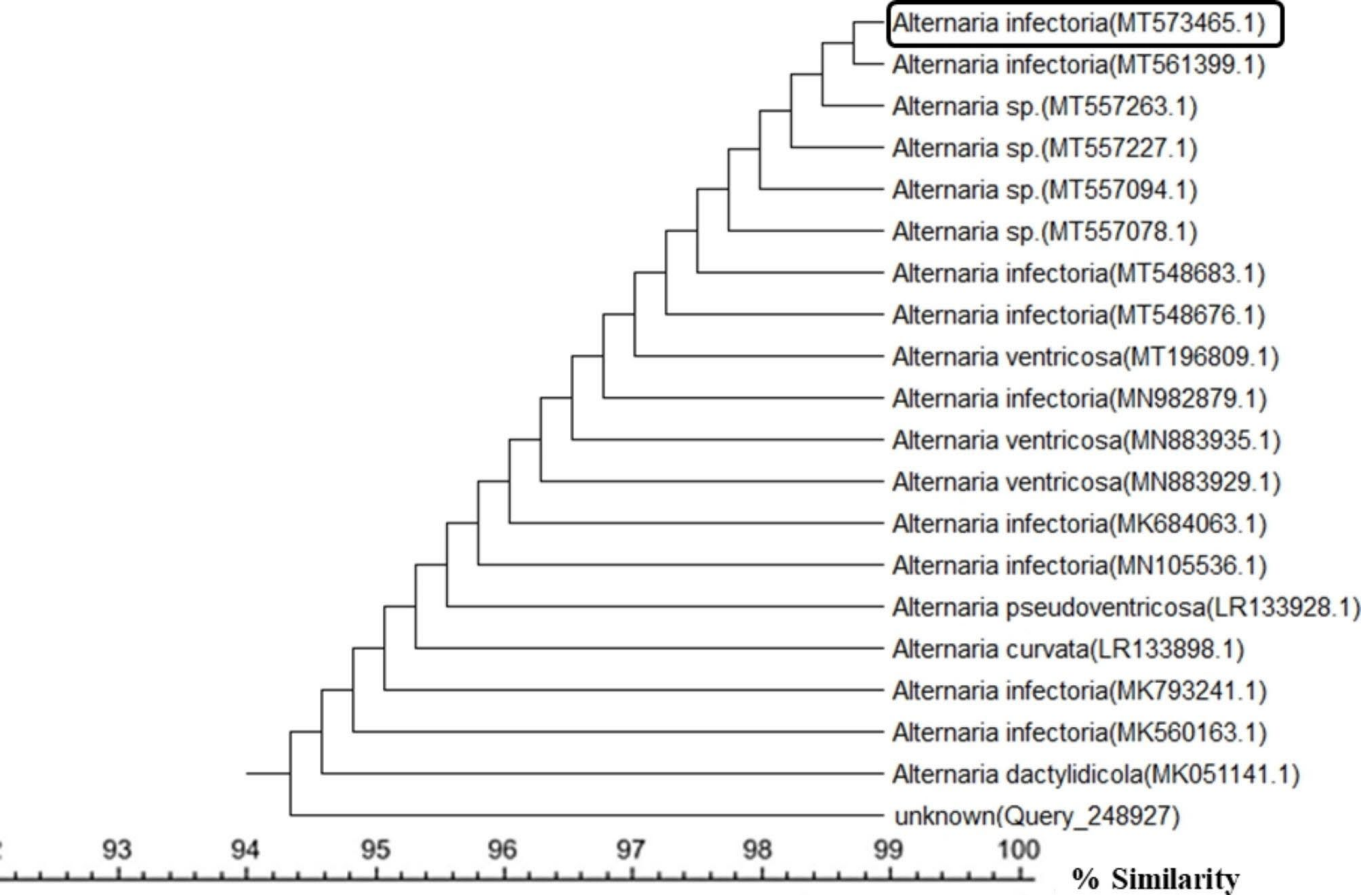
Phylogenetic tree based on neighbour-joining method using MEGA X of the identified endophytic fungus (*Alternaria infectoria* MT573465) isolated from *P. pruinosa* leaves and related sequences

**Fig. 4:**
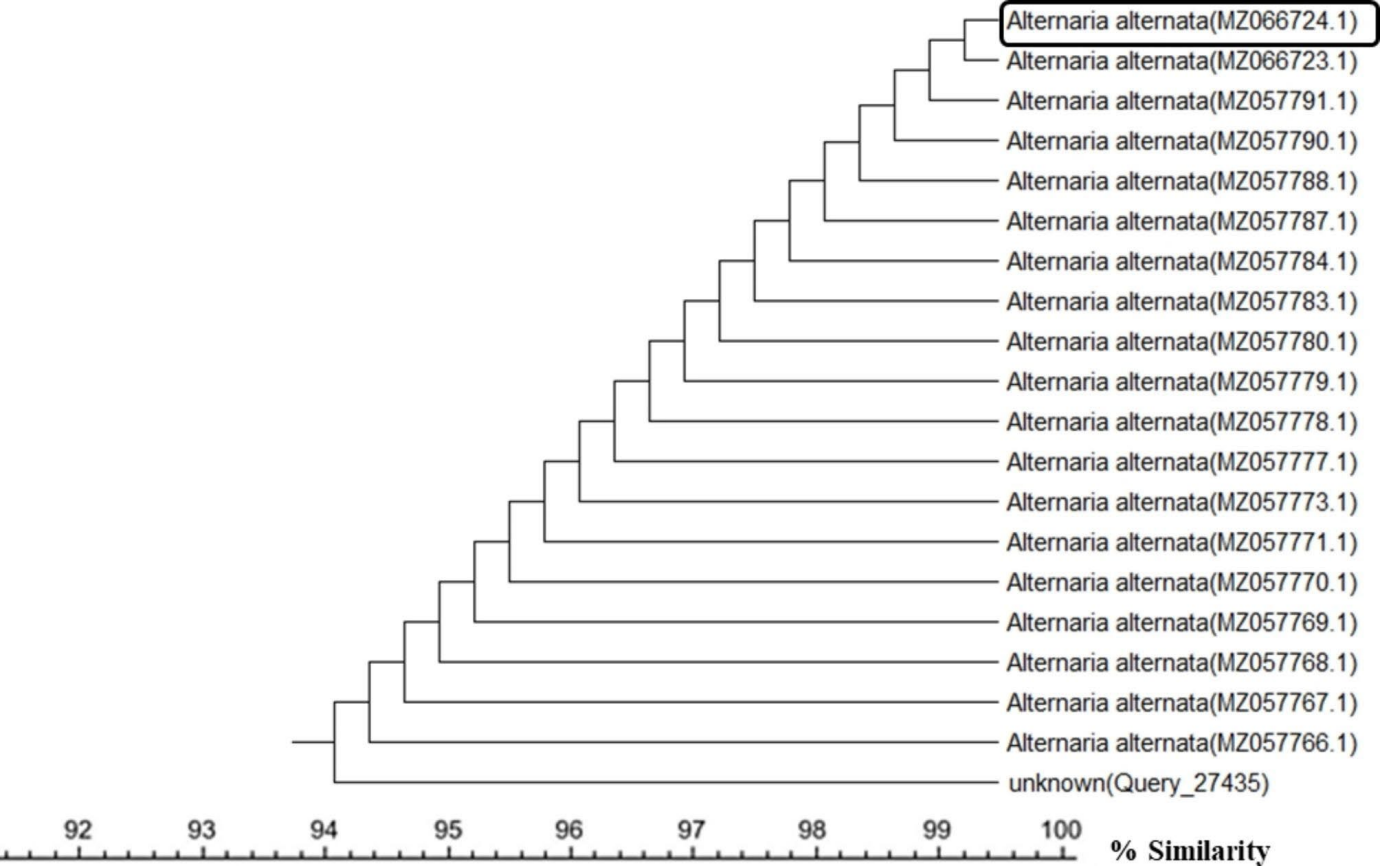
Phylogenetic tree based on neighbour-joining method using MEGA X of the identified endophytic fungus (*Alternaria alternata* MZ066724) isolated from *P. pruinosa* leaves and related sequences

**Fig. 5:**
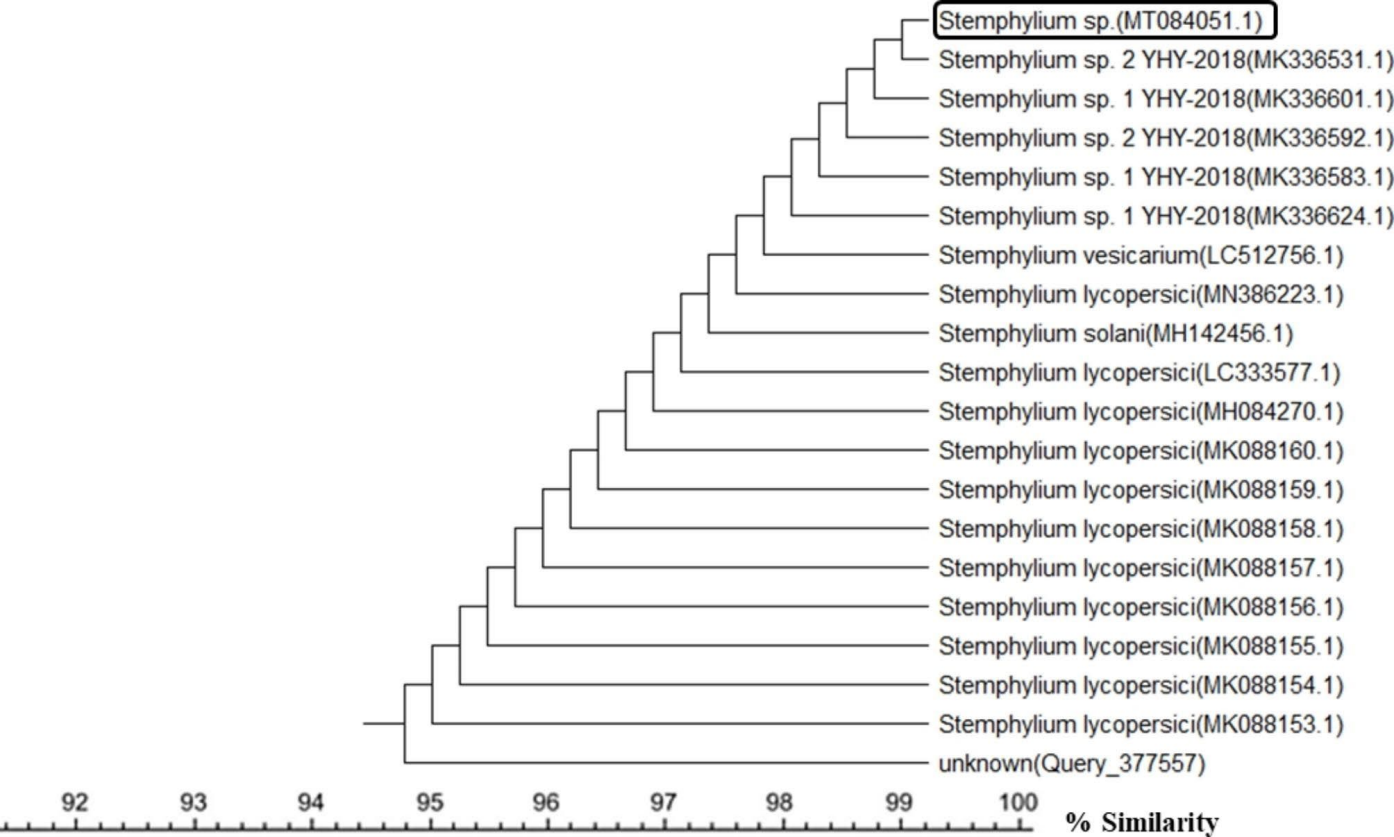
Phylogenetic tree based on neighbour-joining method using MEGA X of the identified endophytic fungus (*Stemphylium sp*. MT084051) isolated from *P. pruinosa* leaves and related sequences

**Fig. 6:**
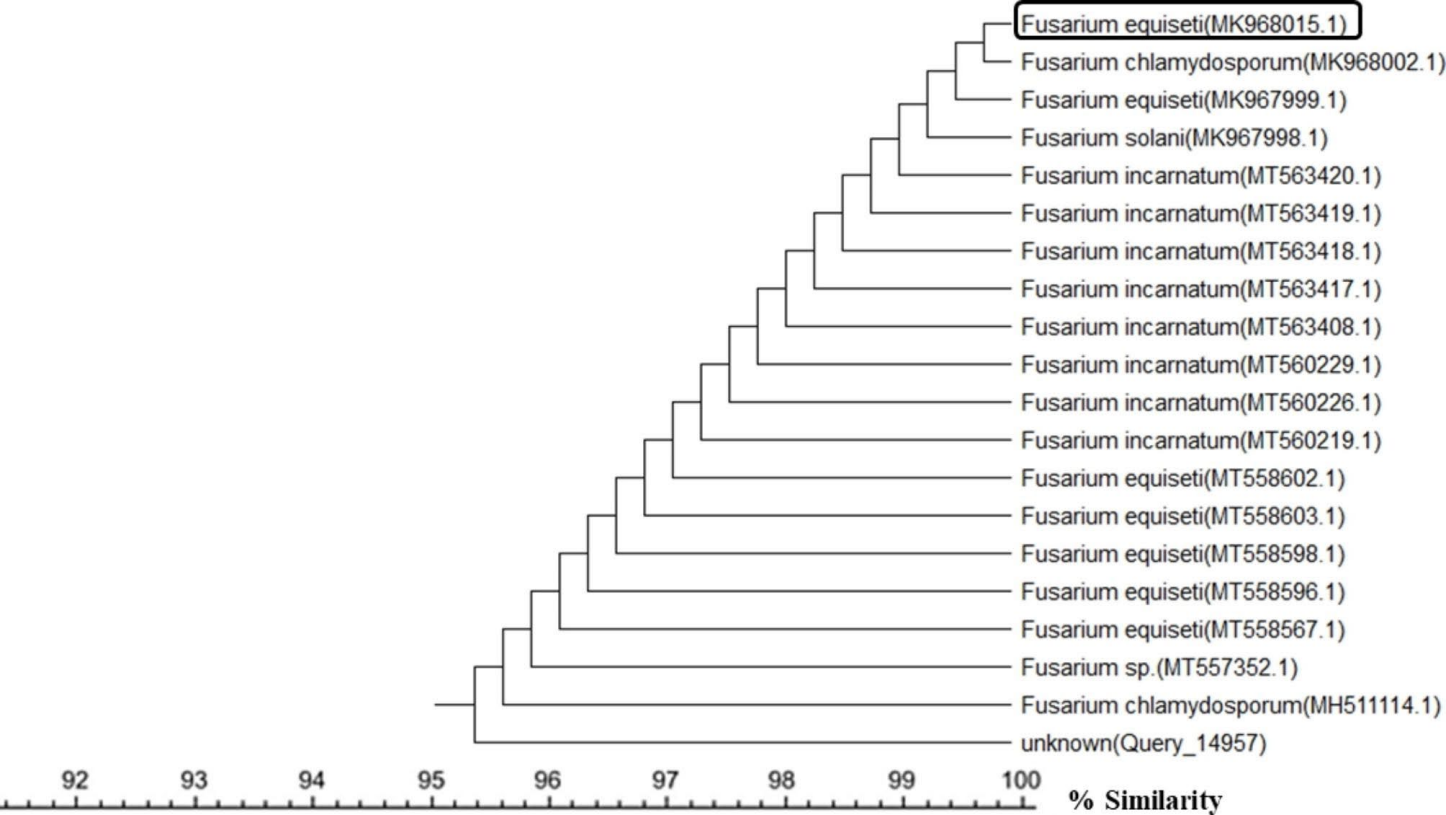
Phylogenetic tree based on neighbour-joining method using MEGA X of the identified endophytic fungus (*Fusarium equiseti* MK968015) isolated from *P. pruinosa* leaves and related sequences

**Fig. 7:**
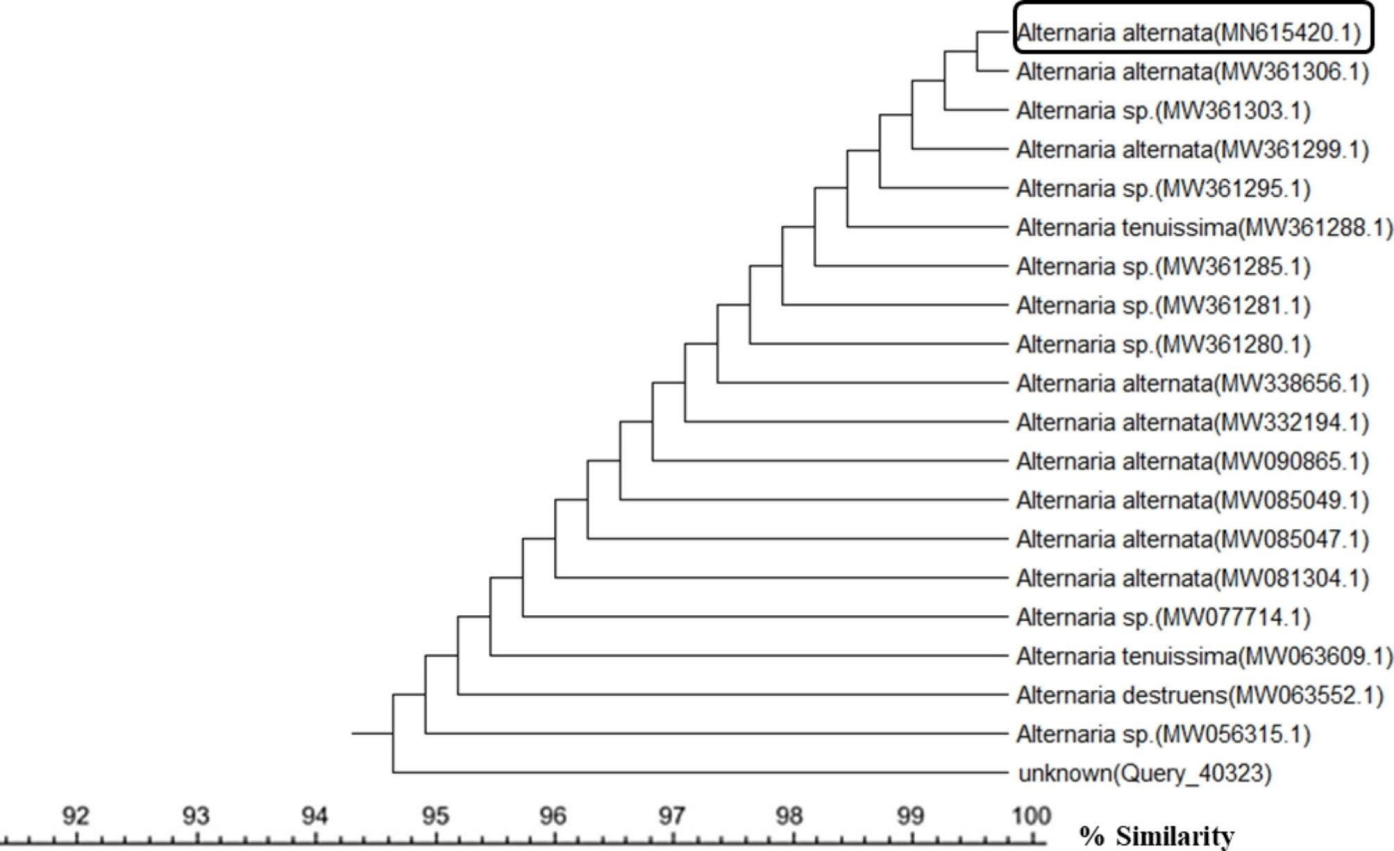
Phylogenetic tree based on neighbour-joining method using MEGA X of the identified endophytic fungus (*Alternaria alternata* MN615420) isolated from *P. pruinosa* leaves and related sequences

A total of 50 endophytic fungal isolates were separated from leaves of *P. pruinosa*. Selection of six representative isolates was performed for further bioactivity screening based on their morphological characters (Fig. 1). Identification of endophytic isolates was done using light microscope by their sporulation structures on PDA medium as *Alternaria sp*., *Stemphylium sp*. And *Fusarium sp*. Molecular identification of the species was detected depending on the score of best sequence alignment and is available under the accession numbers *Stemphylium simmonsii* MN401378 (percent identity, 99.63%), *Alternaria infectoria* MT573465 (percent identity, 98.91%), *Alternaria* alternata MZ066724 (percent identity, 99.21%), *Stemphylium sp*. MT084051 (percent identity, 99.15%), *Fusarium equiseti* MK968015 (percent identity, 99.74%) and *Alternaria alternata* MN615420 (percent identity, 99.54%). The isolated endophytic fungi were represented in the phylogenetic trees as shown in Figs. 2, 3, 4, 5, 6 and 7.

Ethnopharmacologically important medicinal plants are considered as reliable sources for the isolation of endophytes which produce secondary metabolites with unique and therapeutically relevant bioactivities. Endophytes have already been investigated in a variety of plants, but this is the first report of the anti-inflammatory potential of the endophytic fungi associated with *P. pruinosa.* Among the identified isolates, ethyl acetate extract prepared from *A. alternata* MN615420 strain exhibited the greatest anti-inflammatory activity. The obtained results suggested that *A. alternata* MN615420 strain holds bioactive compounds with potential anti-inflammatory activity. Thus, guided isolation of the bioactive compounds from *A. alternata* MN615420 strain was employed.

### Isolation and structural elucidation

**Fig. 8:**
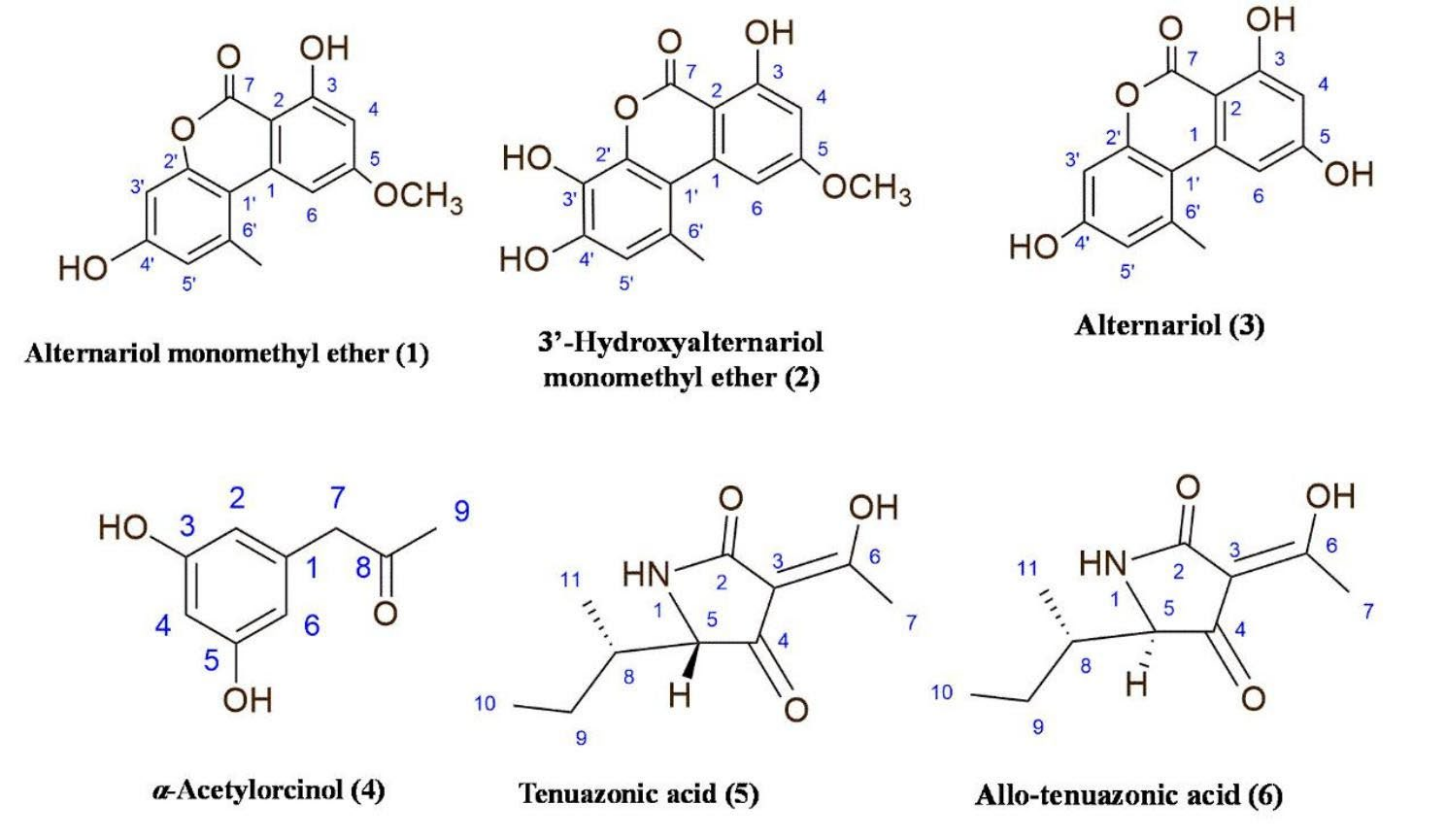
Structures of compounds (1–6) isolated from *A. alternata* MN615420

Six secondary metabolites, including three dibenzo-*α*-pyrone derivatives (**1**–**3**), one resorcinol derivative (**4**) and two tetramic acid derivatives (**5** and **6**) were isolated from *A. alternata* MN615420 ethyl acetate extract (Fig. 8). The metabolites were identified as alternariol monomethyl ether (**1**) [37]’ 3’-hydroxyalternariol monomethyl ether (**2**) [37], alternariol (**3**) [38], *α*-acetylorcinol (**4**) [39] in addition to an inseparable mixture of tenuazonic acid (**5**) and allo-tenuazonic acid (**6**) [40], which were isolated in a ratio (1:1). Elucidation of their chemical structures was based on physicochemical properties in addition to spectral data using 1D and 2D nuclear magnetic resonance (NMR) and MS with electrospray ionization (ESI), as well as by comparison of their properties and spectral characteristics with those in literature. Details of the identification are given under supplementary material (Figures S1-S23).

### Cytotoxicity and anti-inflammatory activities of the identified endophytes and the isolated compounds

It is well known that inflammatory triggers, including immune stimulatory lipopolysaccharides, cause macrophages to release a variety of pro-inflammatory mediators. The activated macrophages produce the pro-inflammatory cytokine interleukin-1beta (IL-1*β*), which upregulates tumor necrosis factor-alpha (TNF-*α*) release that included in various inflammatory disorders. The etiology of autoimmune and chronic inflammatory illnesses has been linked to an increased release of gamma interferon (INF-*γ*). Using the MTT test, safety of the tested extracts and isolated compounds was evaluated and EC100 values that result in 100% cell viability were computed for the tested extracts (Table 1), isolated compounds (Table 2) and the standard anti-inflammatory, piroxicam (Tables 1 and 2). Piroxicam possessed EC100 value of 17.58 ± 2.69 µg/mL. *A. alternata* MN615420 extract showed the highest EC100 value (27.83 ± 2.33 µg/mL) followed by extracts prepared from *A. alternata* MZ066724, *A. infectoria* MT573465, *F. equiseti* MK968015, *Stemphylium sp*. MT084051 and *S. simmonsii* MN401378 as they exhibited EC100 values of 20.38 ± 0.98 µg/mL, 18.99 ± 1.02 µg/mL, 9.76 ± 1.65 µg/mL, 7.42 ± 0.89 µg/mL and 5.97 ± 0.58 µg/mL, respectively. According to these findings, *Alternaria* species had a superior safety profile. Among the isolated compounds, compound 1 (alternariol monomethyl ether) showed the highest EC100 value (77.0 ± 6.0 µM) followed by compound 3 (alternariol, 75.0 ± 5.0 µM). While the inseparable mixture (tenuazonic acid (5) and allo-tenuazonic acid (6)) was the most toxic as it possessed the lowest EC100 value of 8.0 ± 0.1 µM. This information came in accordance with a previous study which considered tenuazonic acid as the most toxic among Alternaria mycotoxins [38].

**Table 1:** Cytotoxicity EC100 values (µg/mL), effective anti-inflammatory concentrations (µg/mL) and TNF-*α* (µg/mL), INF-*γ* (µg/mL) and IL-1*β* (µg/mL) levels of the tested extracts

Sample	Cytotoxicity EC100 (µg/mL) ± SD	Effective anti-inflammatory concentrations (µg/mL) ± SD	TNF-*α* (µg/mL) ± SD	INF-*γ* (µg/mL) ± SD	IL-1*β* (µg/mL) ± SD
*Alternaria alternata* MN615420	27.83 ± 2.33	0.20 ± 0.003	0.20 ± 0.004	0.18 ± 0.008	0.12 ± 0.008
*Alternaria alternata* MZ066724	20.38 ± 0.98	0.54 ± 0.06	0.42 ± 0.08	0.36 ± 0.01	0.40 ± 0.02
*Alternaria infectoria* MT573465	18.99 ± 1.02	0.33 ± 0.01	0.30 ± 0.04	0.15 ± 0.009	0.24 ± 0.03
*Stemphylium sp*. MT084051	7.42 ± 0.89	0.34 ± 0.02	0.28 ± 0.03	0.19 ± 0.007	0.11 ± 0.007
*Stemphylium simmonsii* MN401378	5.97 ± 0.58	0.55 ± 0.07	0.49 ± 0.04	0.49 ± 0.02	0.37 ± 0.05
*Fusarium equiseti* MK968015	9.76 ± 1.65	0.51 ± 0.06	0.41 ± 0.05	0.24 ± 0.01	0.29 ± 0.06
Piroxicam	17.58 ± 2.69	1.01 ± 0.25	0.57 ± 0.01	1.36 ± 0.30	0.66 ± 0.10
LPS-stimulated WBCs	-	-	2.36 ± 0.85	5.69 ± 1.02	2.69 ± 0.58
Normal cells (control)	-	-	0.79 ± 0.03	2.51 ± 0.58	1.16 ± 0.085

Values are expressed as mean ± SD, (n = 3).

**Table 2:** Cytotoxicity EC100 values (µM), effective anti-inflammatory concentrations (µM) and TNF-*α* (µg/mL), INF-*γ* (µg/mL) and IL-1*β* (µg/mL) levels of the isolated compounds

Sample	Cytotoxicity EC100 (µM) ± SD	Effective anti-inflammatory concentrationS (µM) ± SD	TNF-*α* (µg/mL) ± SD	INF-*γ* (µg/mL) ± SD	IL-1*β* (µg/mL) ± SD
Compound **1** Alternariol monomethyl ether	77.0 ± 6.0	1.0 ± 0.3	0.25 ± 0.02	0.27 ± 0.06	0.30 ± 0.03
Compound **2** 3’-Hydroxyalternariol monomethyl ether	64.0 ± 3.0	0.9 ± 0.2	0.31 ± 0.09	0.20 ± 0.07	0.18 ± 0.004
Compound **3** Alternariol	75.0 ± 5.0	2.0 ± 0.03	0.27 ± 0.08	0.34 ± 0.02	0.24 ± 0.009
Compound **4** *α*-Acetylorcinol	20.0 ± 2.0	3.7 ± 0.06	0.37 ± 0.009	0.39 ± 0.01	0.21 ± 0.08
Mixture of tenuazonic acid (compound **5**) and allo-tenuazonic acid (compound **6**)	8.0 ± 1.0	3.8 ± 0.1	0.34 ± 0.007	0.34 ± 0.05	0.23 ± 0.02
Piroxicam	53.0 ± 8.0	3.0 ± 0.7	0.57 ± 0.01	1.36 ± 0.30	0.66 ± 0.10
LPS-stimulated WBCs	-	-	2.36 ± 0.85	5.69 ± 1.02	2.69 ± 0.58
Normal cells (control)	-	-	0.79 ± 0.03	2.51 ± 0.58	1.16 ± 0.085

Values are expressed as mean ± SD, (n = 3).

The effective anti-inflammatory concentrations (EAICs) of the studied extracts and isolated compound was then estimated, which defined as the concentrations that could restore the aberrant proliferation of LPS-stimulated cells to the normal proliferation of control. The EAIC values of all extracts were evaluated and were all found to be lower than that of the positive control piroxicam (p < 0.05), thus indicating their efficacy. As concluded from Table 1, *A. alternata* MN615420 extract possessed the lowest EAIC value of 0.20 ± 0.003 µg/mL, while *S. simmonsii* MN401378 extract showed the highest value of 0.55 ± 0.07 µg/mL. Regarding the isolated compounds (Table 2), compound 2 (3’-hydroxyalternariol monomethyl ether) was found to be the most potent anti-inflammatory candidate relative to piroxicam as it possessed the lowest EAIC value of 0.9 ± 0.2 µM, p *<* 0.05.

Gene expression of the three pro-inflammatory indicators (TNF-*α*, IL-1*β*, INF-*γ*) was examined in order to determine the mechanism of anti-inflammatory activity of tested extracts and isolated compounds by real time polymerase chain reaction (PCR) in normal WBCs and the treated WBCs with lipopolysaccharide (LPS). The cell wall of gram-negative bacteria contains proteins known as lipopolysaccharides (LPS), which break down into O-antigen, lipid-A and core protein. Lipid-A is known to have strong immunogenic and pro-inflammatory effects. Pro-inflammatory cytokines such as TNF-*α*, IL-1*β* and INF-*γ* play crucial roles in immune system and inflammation process like vasodilatation and edema. In addition to their key role in chronic inflammation [41]. The studied extracts reduced the gene upregulation of the evaluated cytokines in LPS-treated WBCs to levels lower than those caused by LPS as shown in Table 1. It could also be observed that *A. alternata* MN615420 extract was the most potent inhibitor of TNF-*α* with reducing the concentration to 0.20 ± 0.004 µg/mL. While *A. infectoria* MT573465 extract was the most potent INF-*γ* inhibitor with a reduction of its concentration to 0.15 ± 0.009 µg/mL. *Stemphylium sp*. MT084051 extract significantly reduced the elevated level of IL-1*β* to a value of 0.11 ± 0.007 µg/mL; compared to the value obtained with the positive control piroxicam, this value was significantly lower (0.66 ± 0.10 µg/mL, p < 0.05). Among the tested isolated compounds (Table 2), compound 2 (3’-hydroxyalternariol monomethyl ether) showed the highest anti-inflammatory potential with the most considerable reductions in the level of INF-*γ* (0.20 ± 0.07 µg/mL) and IL-1*β* (0.18 ± 0.004 µg/mL). While compound 1 (alternariol monomethyl ether) was the most potent TNF-*α* inhibitor with a reduction of its concentration to 0.25 ± 0.02 µg/mL.

These outcomes were consistent with previous studies which evaluated the anti-inflammatory impact of *Alternaria* species. For instance, a previous study demonstrated that fractions of *A. alternata* exhibited an in vitro anti-inflammatory activity *via* inhibition of heat induced albumin denaturation besides red blood cells membrane stabilization [42].

### Molecular docking study

The present study included a molecular docking analysis to investigate the conformational space of the ligand within the protein-binding site and predict the complex structure created by ligand-protein binding. The energy values for the protein (IL-1*β*, TNF-*α* and INF-*γ*)–ligand interaction for the best conformation of piroxicam (the standard anti-inflammatory drug) and the isolated compounds were estimated (Table 3).

**Table 3:** Docking scores (kcal/mol) of TNF-*α*, IFN-*γ* and IL-1*β* with the isolated compounds and the reference anti-inflammatory drug (piroxicam)

	TNF-*α* (2AZ5)	IFN-*γ* (6E3K)	IL-1*β* (4DEP)
**Compound 1** Alternariol monomethyl ether	-7.648	-4.682	-2.830
**Compound 2** 3’-Hydroxyalternariol monomethyl ether	-6.274	-5.242	-4.452
**Compound 3** Alternariol	-6.414	-4.246	-3.788
**Compound 4** *α*-Acetylorcinol	-5.054	-3.989	**-**2.931
**Compound 5** Tenuazonic acid	-4.593	-2.845	-3.121
**Compound 6** Allo-tenuazonic	-4.487	2.845	-3.038
Piroxicam	-4.800	-2.982	-2.841

#### Molecular docking of IL-1β with the isolated compounds

**Fig. 9:**
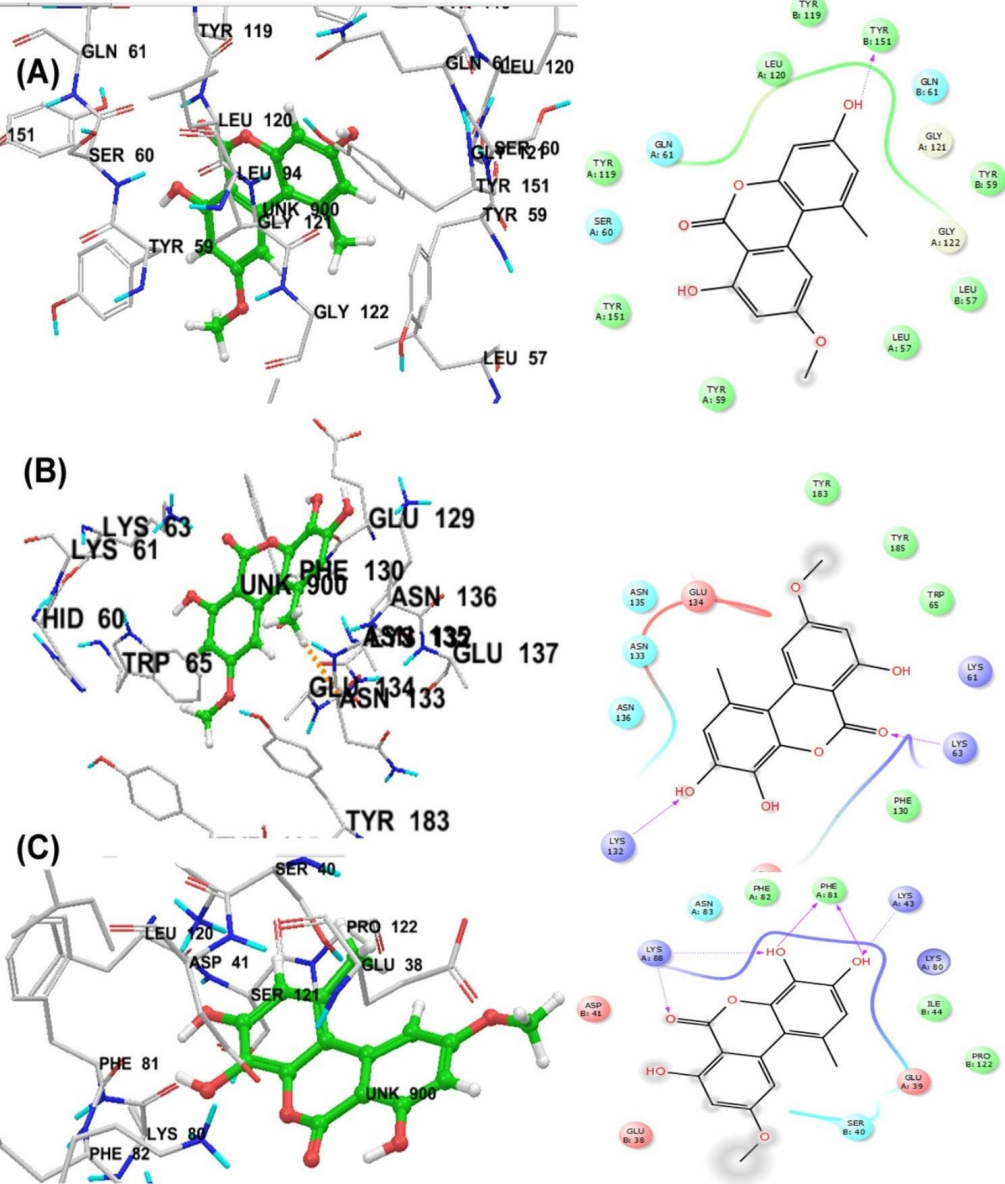
(**A**) 2D and 3D interaction diagrams of alternariol monomethyl ether in the active site of TNF-*α* (2AZ5). (**B**) 2D and 3D interaction diagrams of 3’-hydroxyalternariol monomethyl ether in the active site of IL-1*β* (4DEP). (**C**) 2D and 3D interaction diagrams of 3’-hydroxyalternariol monomethyl ether in the active site of IFN-*γ* (6E3K)

Interleukine-1 is considered one of the most important families of cytokines, which consists of 11 members. This family is known as endogenous pyrogens and is implicated in pro-inflammatory reactions. The members of this family bind to a class of receptor known as IL-1R. There are two receptors for IL-1, IL-1 type 1 receptor (IL-1RI) and IL-1 type 2 receptor (IL-1RII). The two most significant IL-1 cytokine members are IL-1*α* and IL-1*β*. To carry out their respective functions, IL-1*α* and IL-1*β* bind to the same receptor (IL-1RI). A natural antagonist, the protein IL-1ra, binds to IL-1RI and prevents IL-1 from interacting with the receptor. IL-1*β* has a superior importance owing to its vital role in various immune responses in the body [34]. In the current study, majority of the evaluated compounds displayed favorable docking scores when compared to the standard reference ligand (docking score of piroxicam = − 2.84 kcal/mol), with the best-scored ligands being compound **2** (3’-hydroxyalternariol monomethyl ether, docking score = − 4.45 kcal/mol) followed by compound **3** (alternariol, docking score = − 3.78 kcal/mol) (Table 3). The best docked conformation of compound **2** (Fig. 9) revealed the interaction of the ketonic oxygen with the side chain amino group of Lys63. In addition, the phenolic group in ring A mediated hydrogen bond interaction with the side chain amino group of Lys132 [43]. C-ring of compound **2** was stabilized by hydrophobic interactions provided by the side chains of the surrounding residues. Moreover, the bound ligand (compound **2**) was stabilized by a number of polar interactions with the following residues: Asn133, Asn135 and Asn136. All these interactions contributed significantly to the superior stability of compound **2** (3’-hydroxyalternariol monomethyl ether) as a ligand. The docking scores and the experimental outcomes showed a very good match. As compound 2 (3’-hydroxyalternariol monomethyl ether) showed the highest anti-inflammatory potential with the most considerable reduction in the level of IL-1*β.*

#### Molecular docking of TNF-α with the isolated compounds

TNF-*α* is a pro-inflammatory cytokine with a key role in apoptosis, inflammation, proliferation, differentiation and immune regulation. TNF-*α* plays a significant part in the inflammatory process, hence therapeutic research on drugs that block its action has gained a lot of attention recently [44]. The isolated alternariol derivatives exhibited good inhibitory potential against TNF-*α* with docking scores − 7.64 kcal/mol for compound **1** (alternariol monomethyl ether), − 6.41 kcal/mol for compound **3** (alternariol) and − 6.27 kcal/mol for compound **2** (3’-hydroxyalternariol monomethyl ether) as illustrated in Table 3. Detailed binding mode analysis demonstrated that alternariol derivatives mediated a similar form of interactions as observed in a previous study [45]. Detailed common molecular interaction pattern for the three compounds (presented by compound 1, Fig. 9) demonstrated that ring A phenolic group established hydrogen bond interaction with the side chain of Tyr151. The hydrophobic cleft formed by the crucial residues of TNF-α (Leu120, Tyr59, Tyr119 and Tyr151) around the top scoring alternariol derivatives provided additional stability to better fit the ligands in the active site of TNF-*α*. Thus, suggested that alternariol derivatives could be considered as direct inhibitors of the target protein as verified by the experimental results.

#### Molecular docking of INF-γ with the isolated compounds

IFN-*γ* is a well-known proinflammatory cytokine with a prominent function in inflammation and autoimmune diseases. The three isolated alternariol derivatives (compounds **1**–**3**) were more active as compared to the reference anti-inflammatory drug, piroxicam (docking score = − 2.98 kcal/mol) with compound **2** (3’-hydroxyalternariol monomethyl ether) being the most active (docking score = − 5.24 kcal/mol) as shown in Table 3. The binding mode analysis of compound **2** revealed four hydrogen bond contacts between the phenolic groups in ring A and PheA81, LysA43 and LysA88. Also, the ketonic group established hydrogen bond interaction with the side chain of LysA88 (Fig. 9). Moreover, compound **2** showed hydrophobic interactions along with the hydrogen bond contacts with the vital residues of IFN-*γ*. Thus, 3’-hydroxyalternariol monomethyl ether was presumed to be a direct inhibitor of the target protein. An excellent correlation was obtained between the docking scores and the experimental results as compound 2 (3’-hydroxyalternariol monomethyl showed the most considerable reductions in the level of INF-*γ*. The docking outcomes demonstrated the existence of important binding interactions between protein-ligand complexes. This study opens new avenues for the design and development of innovative anti-inflammatory candidates that specifically target INF-*γ*, IL-1*β* and INF-*γ*.

## Conclusions

In this work, isolation for the first time some endophytic fungi from the medicinal plant *P. pruinosa* was successfully attempted. Moreover, comparative evaluation of ex vivo anti-inflammatory activity in addition to cytokines gene regulation of the identified endophytic fungi on LPS-stimulated white blood cells was endeavored followed by isolation of the main active metabolites from the most potent anti-inflammatory candidate. Assessment of ex vivo anti-inflammatory activity of the isolated compounds as well as the mechanisms underlying these effects using computer-aided molecular docking was performed. The six selected isolated endophytic fungi were identified by both morphological and molecular methods as *Stemphylium simmonsii* MN401378, *Stemphylium sp*. MT084051, *Alternaria infectoria* MT573465, *Alternaria alternata* MZ066724, *Alternaria alternata* MN615420 and *Fusarium equiseti* MK968015. Six secondary metabolites, including three dibenzo-*α*-pyrone derivatives (1–3), one resorcinol derivative (4) and two tetramic acid derivatives (5 and 6) were isolated from *A. alternata* MN615420 ethyl acetate extract. It could be observed that *A. alternata* MN615420 extract was the most potent anti- inflammatory candidate with the most significant downregulation of TNF-*α* level. While *A. infectoria* MT573465 extract was the most potent INF-*γ* inhibitor. Moreover, *Stemphylium sp*. MT084051 extract significantly reduced the elevated level of IL-1*β*. Among the tested isolated compounds, compound 2 (3’-hydroxyalternariol monomethyl ether) showed the highest anti-inflammatory potential with the most considerable reductions in the levels of INF-*γ* and IL-1*β*. While compound 1 (alternariol monomethyl ether) was the most potent TNF-*α* inhibitor. The present study included a molecular docking analysis to investigate the conformational space of the ligand within the protein-binding site and predict the complex structure created by ligand-protein binding. The results obtained suggested alternariol derivatives as naturally occurring potent anti-inflammatory candidates. However, further extensive in vivo studies are required to verify the obtained results.

## Electronic supplementary material

Below is the link to the electronic supplementary material.

**Additional file 1**.

## Data Availability

Data is available upon request.

## Abbreviations

LPS: Lipopolysaccharides
TNF-*α*: Tumor necrosis factor-alpha
IFN: Interferons
ILs: Interleukins
PCR: Polymerase chain reaction
PDA: Potato dextrose agar
PDB: Potato dextrose broth
EAICs: Effective anti-inflammatory concentrations
SI: Stimulation index
