# Supplementary material for: Bio-guided isolation of potential anti-inflammatory constituents of some endophytes isolated from the leaves of ground cherry (Physalis pruinosa L.) via ex-vivo and in-silico studies
Source: BMC Complement Med Ther. 2023 Apr 3;23:103. doi: 10.1186/s12906-023-03934-9 (PMC10069101; doi:10.1186/s12906-023-03934-9)
Supplement: Supplementary file 1 — Additional file 1. [file 12906_2023_3934_MOESM1_ESM.docx]

**Bio-guided isolation of potential anti-inflammatory constituents of some endophytes isolated from the leaves of Ground Cherry (*Physalis pruinosa* L.) via *ex-vivo* and *in-silico* studies**

**Asmaa Mahana^1^, Hala M. Hammoda ^1^, Mona M. G. Saad ^2^, Mohamed M. Radwan ^1,3^,****Mahmoud A. ElSohly ^3,4^, Doaa A. Ghareeb ^5,6^, Fathallah M. Harraz ^1^, Eman Shawky ^1*^**

^1^ Department of Pharmacognosy, Faculty of Pharmacy, Alexandria University, Egypt.

^2^ Department of Pesticide Chemistry and Technology, Faculty of Agriculture, 21545-El-Shatby, Alexandria University, Alexandria, Egypt.

^3^ National Center for Natural Products Research, School of Pharmacy, University of Mississippi, University, MS 38677, USA.

^4^ Department of Pharmaceutics and Drug Delivery, University of Mississippi, University, MS 38677, USA.

^5^ Bio-screening and Preclinical Trial Lab, Biochemistry Department, Faculty of Science, Alexandria University, Alexandria, Egypt.

^6^ Biochemistry Department, Faculty of Science, Alexandria University, Alexandria, Egypt.

* Author of correspondence: Dr. Eman Shawky, Alkhartoom square, Department of Pharmacognosy, Faculty of Pharmacy, Alexandria University, Egypt, Alexandria 21521, Egypt.

E-mail: [shawkyeman@yahoo.com](mailto:shawkyeman@yahoo.com), [eman.m.shawky@alexu.edu.eg](mailto:eman.m.shawky@alexu.edu.eg).

**0201005294669**

**Spectral data of alternariol derivatives (compounds 1-3)**

^1^HNMR spectra of compounds **1** to **3** suggested their relation to alternariol metabolite . Compounds **1** and **3** were isolated as violet and colorless crystals, respectively. Their ESI-MS spectra showed deprotonated ion peaks at m/z 271.1 (compound **1**) and 257.0 (compound **3**) (Figures 1S and 2S). Compounds **1** and **3** exhibited a very similar ^1^HNMR data. Two tetrasubstituted aromatic rings were suggested based on the *met*a coupled four doublets detected at the aromatic region (δ_H_ 6.38-7.26). A sharp singlet at δ_H_ 2.75 and 2.71 was detected in compounds **1** and **3**, respectively (integrating for 3H) indicated the presence of a methyl group attached to an aromatic ring. There was an only difference between the ^1^HNMR spectra of both compounds, represented in the existence of another methyl singlet at δ_H_ 3.92 in compound **1**, thus suggesting the presence of a methoxyl group attached to one of the aromatic rings.

^13^CNMR spectra of compounds **1** and **3** are quite similar except for the presence of a carbon signal detected at δ_C_ 56.2, which attributed to a methoxyl group in compound **1**. Both compounds showed similar HMBC correlations (Figures S7 and S11), which supported the existence of alternariol chemical skeleton. H-5' of compound **1** showed correlations with carbon atoms detected at δ_C_ 109.1 and δ_C_ 99.7, which assigned as C-1' and C-3', respectively. Also, H-5' of compound **3** showed correlations with carbon atoms at δ_C_ 102.1 (C-3') and δ_C_ 109.4 (C-1'). Also, the connectivity between both rings B and C in compound **1** can be concluded from the HMBC correlations, as H-6, which detected at δ_H_ 7.25, correlated through three bonds with carbons at δ_C_ 109.1 and δ_C_ 99.0 (C-1' and C-2, respectively). The same HMBC correlations of H-6 (δ_H_ 7.26) with both C-1' (δ_C_ 109.4) and C-2 (δ_C_ 97.8) can be observed for compound **3.** By comparison with previous reports [1][2], compounds **1** and **3** were identified as alternariol monomethyl ether and alternariol, respectively. Alternariol monomethyl ether (**1**) and alternariol (**3**) have been isolated from several cultures belonging to the genus *Alternaria*, including *A. alternata* [1], *A. infectoria* [3] and *A. tenuissima* [4].

Comparing compound **2** to alternariol monomethyl ether (**1**), the ^1^HNMR results showed a striking similarity. The absence of two doublets at δ_H_ 6.74 and 6.64 was the main difference between both compounds. Instead, compound **2** showed a singlet at δ_H_ 6.99, pointing to the possibility of the presence of a new substituent attached to the ring A**.** By comparison with previous reports and the available authentic reference, compound **2** was identified as 3'-hydroxyalternariol monomethyl ether, which was previously isolated from several cultures belonging to the genus *Alternaria* [1]*.*


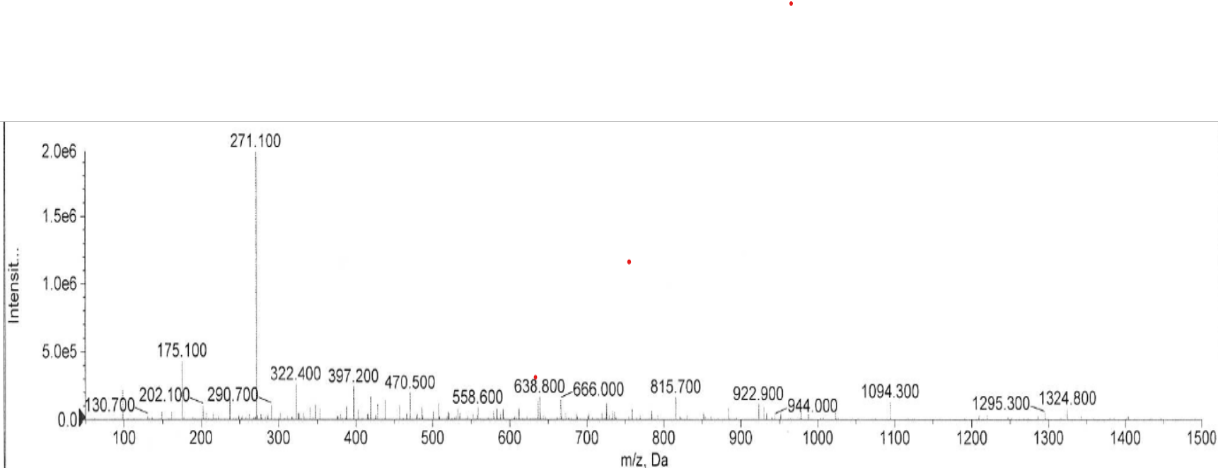


**Figure S1: ESI-MS spectrum of compound 1.**


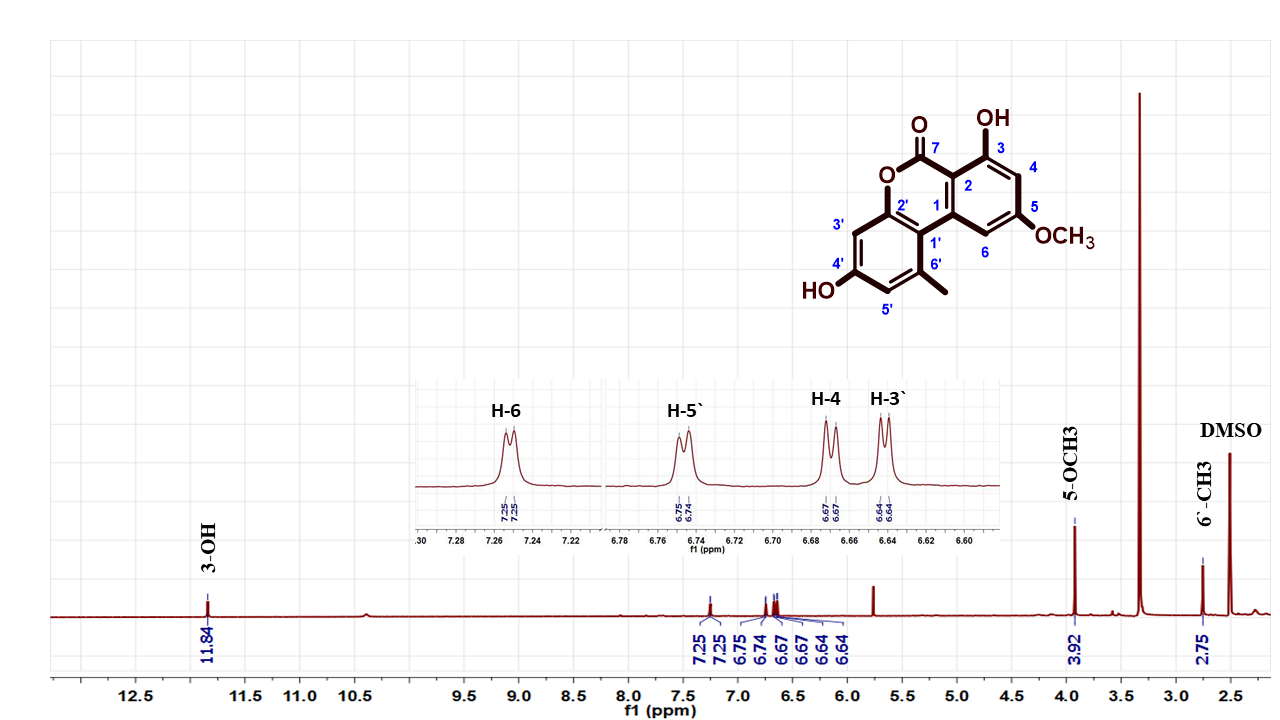


**Figure S2: ^1^HNMR spectrum of compound 1.**


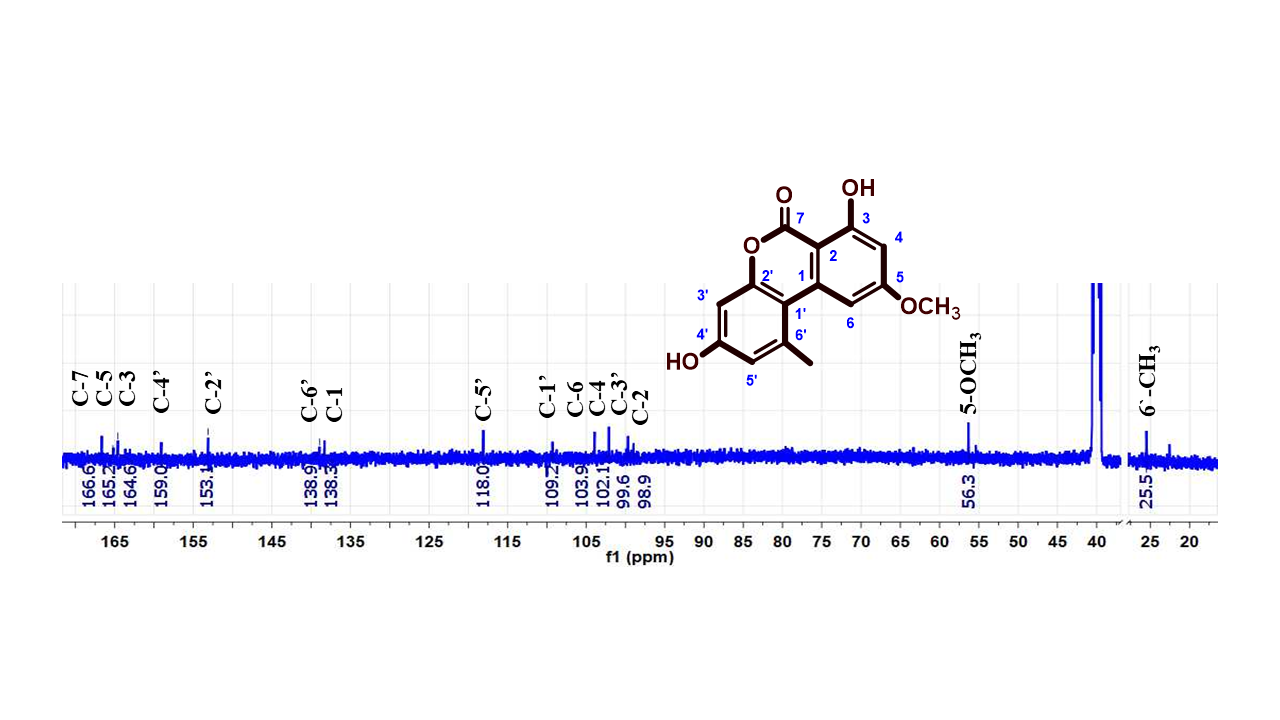


**Figure S3: ^13^CNMR spectrum of compound 1.**


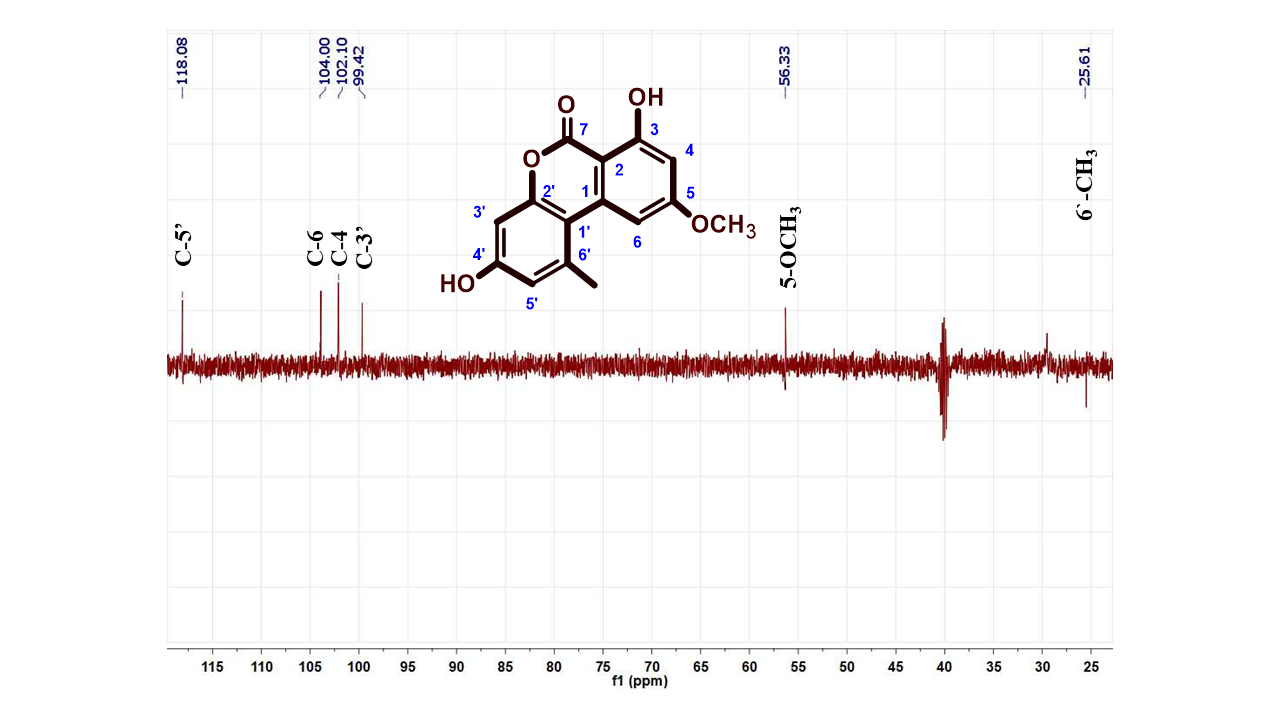


**Figure S4: DEPT-135 spectrum of compound 1.**


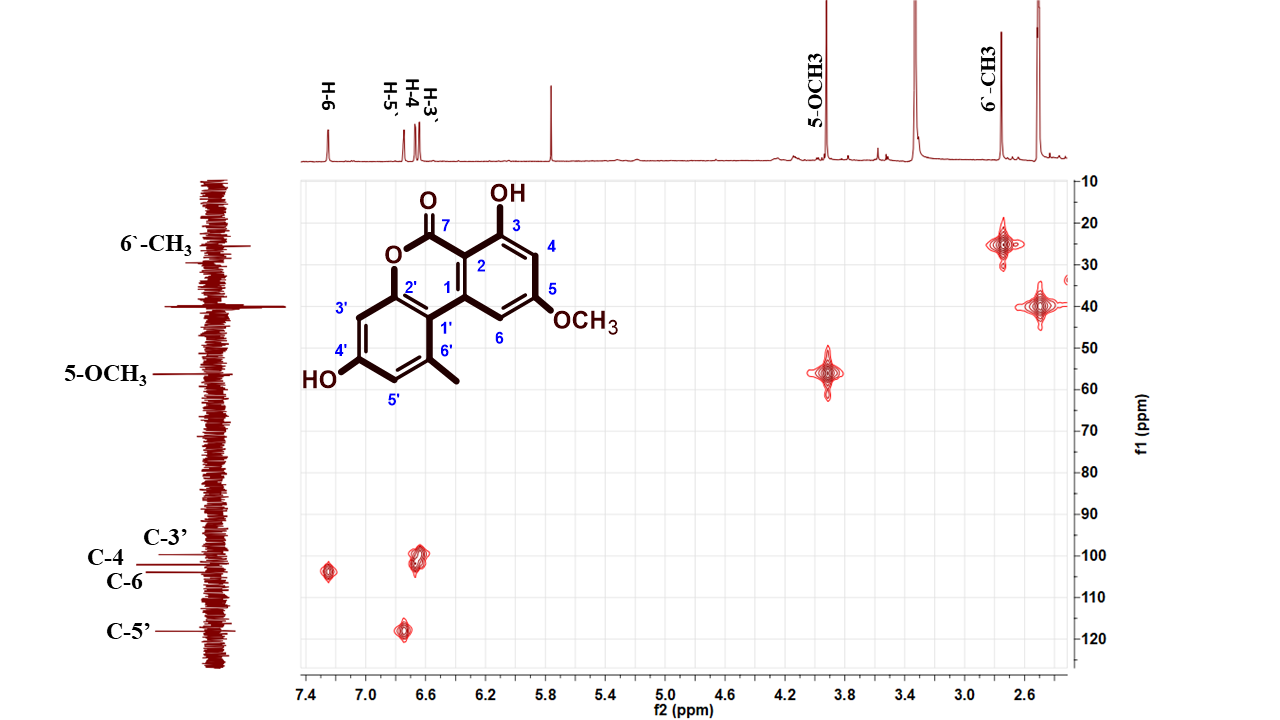
**Figure S5: HMQC spectrum of compound 1.**


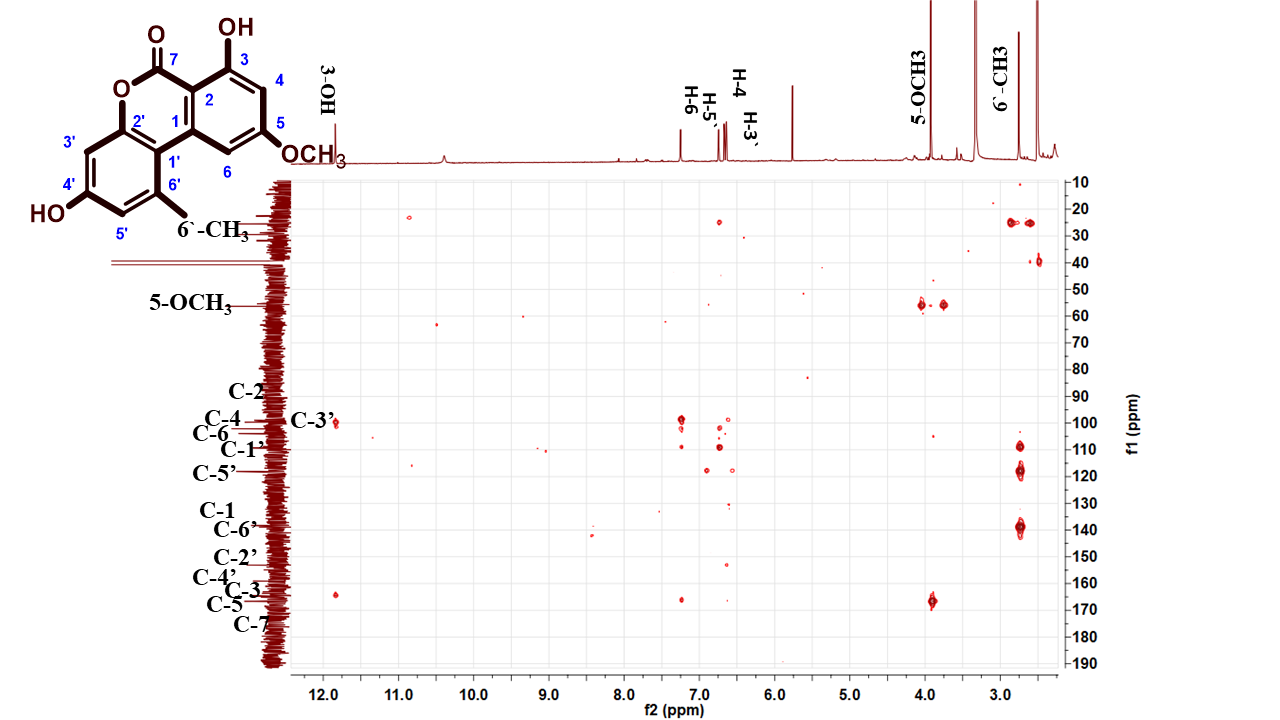
**Figure S6: HMBC spectrum of compound 1.**

**
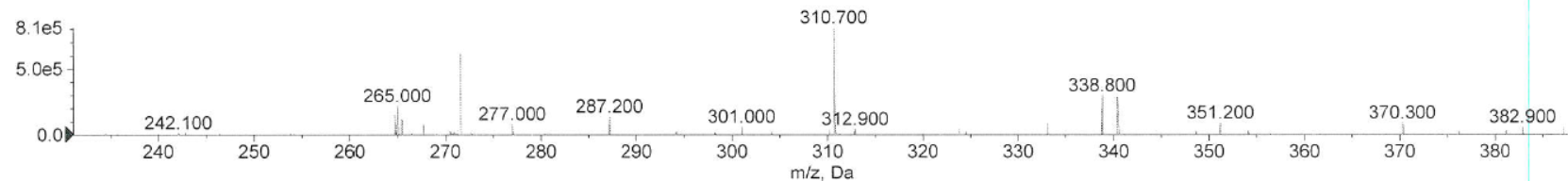
**

**Figure S7: ESI-MS spectrum of compound 2.**


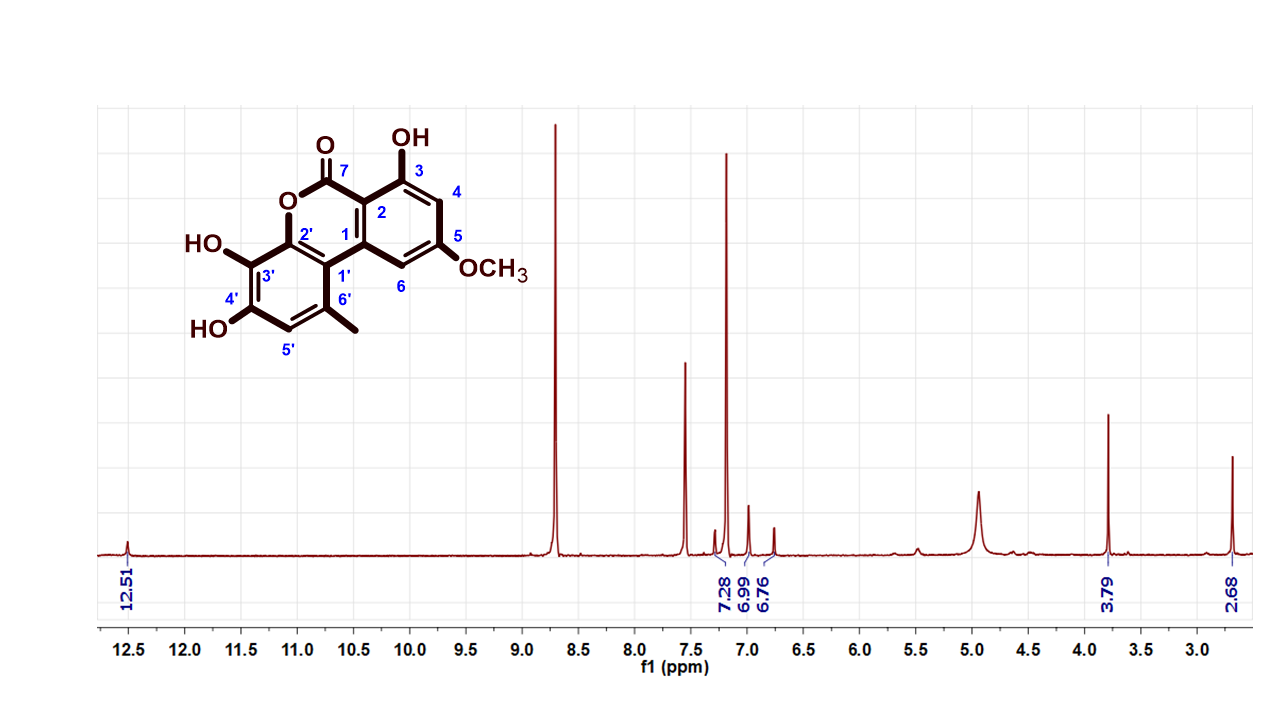
**Figure S8: ^1^HNMR spectrum of compound 2.**

**
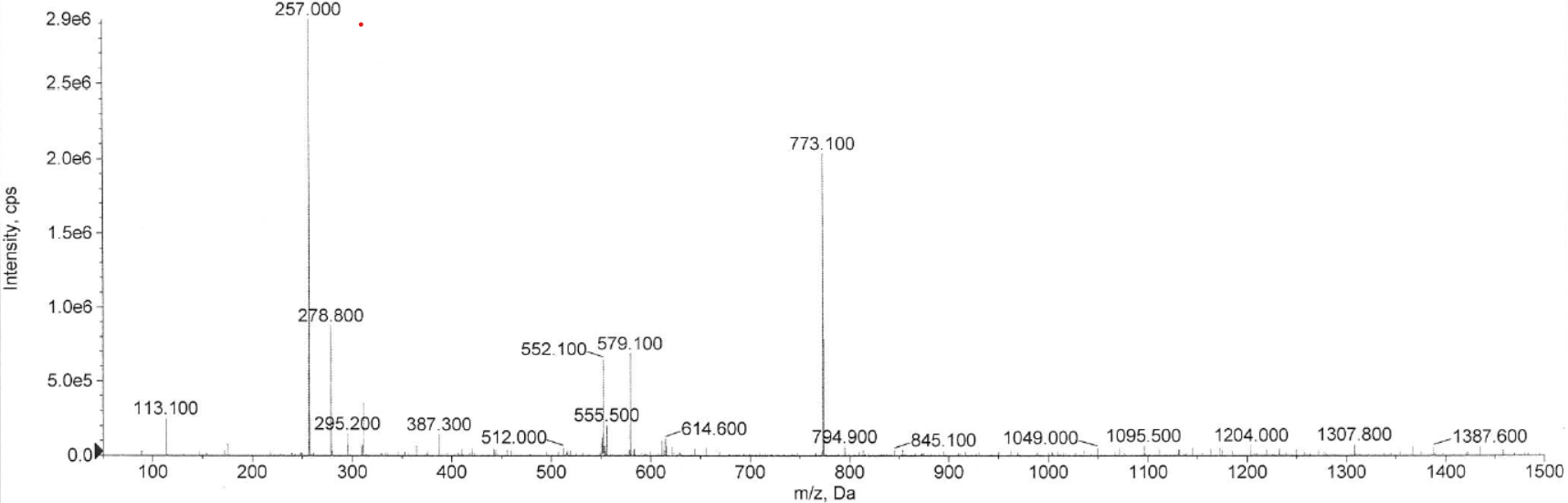
**

**Figure S9: ESI-MS spectrum of compound 3.**


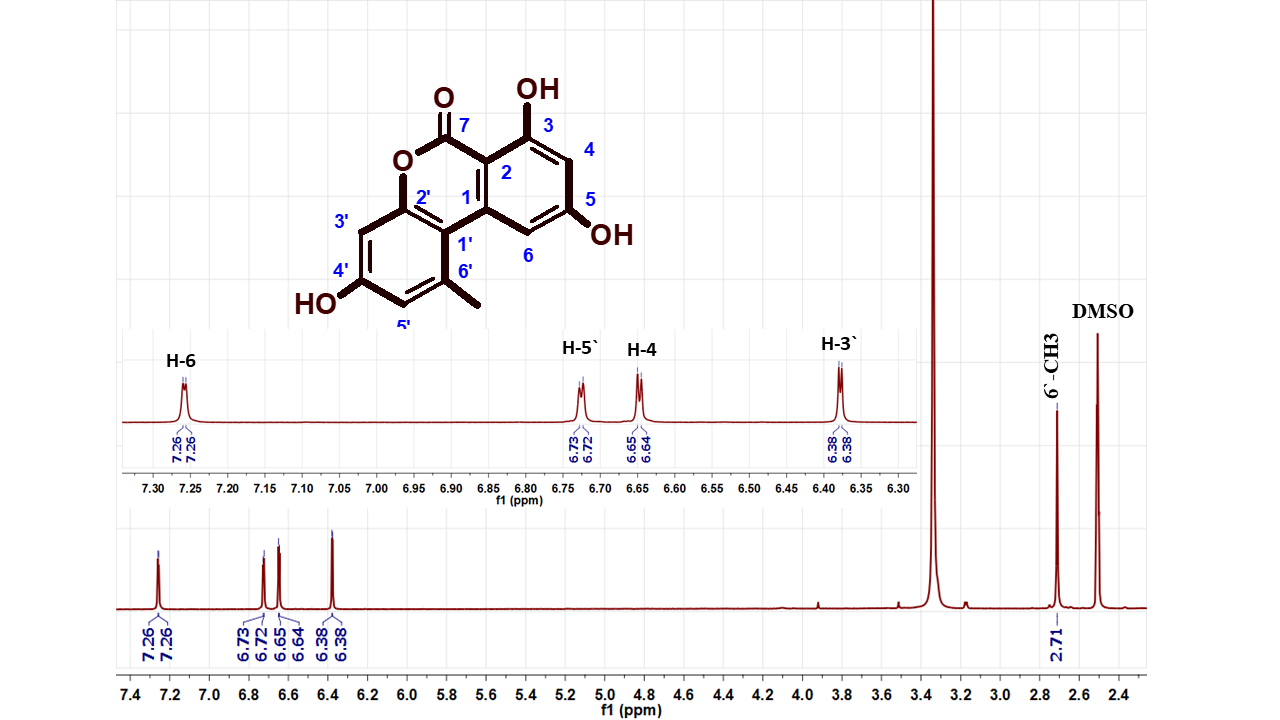
**Figure S10: ^1^HNMR spectrum of compound 3.**


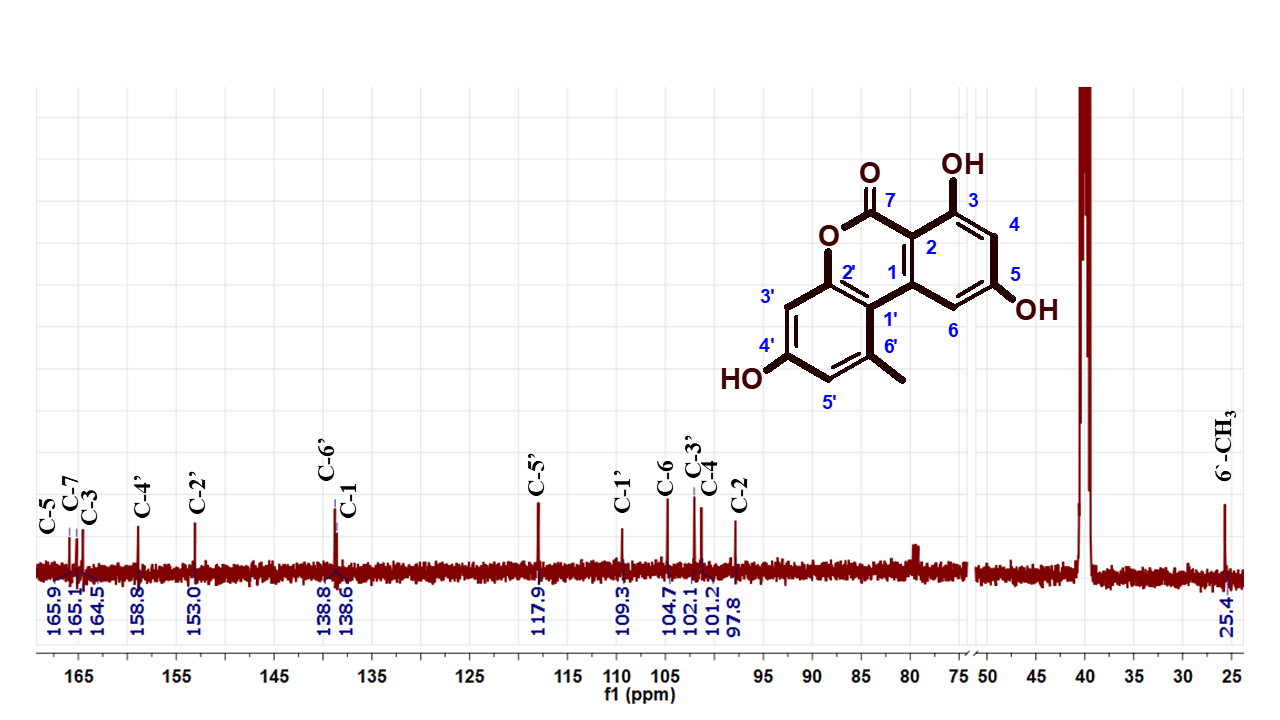
**Figure S11: ^13^CNMR spectrum of compound 3.**


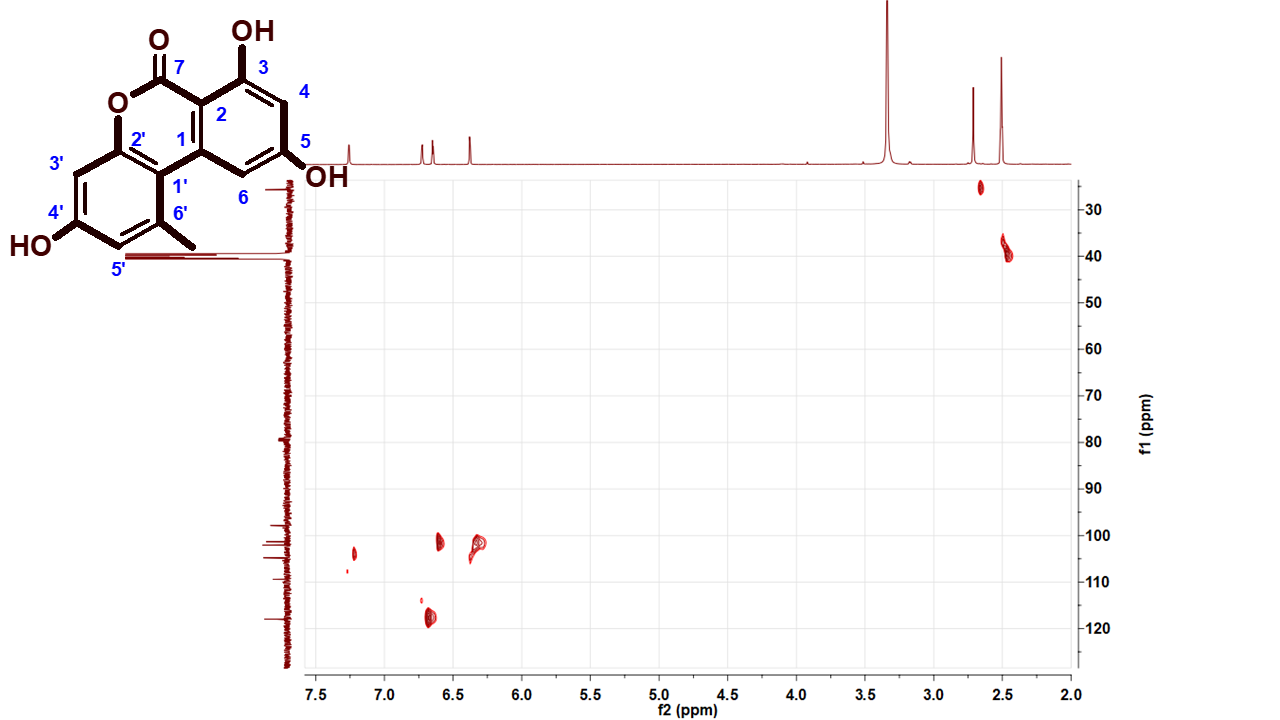
**Figure S12: HMQC spectrum of compound 3.**


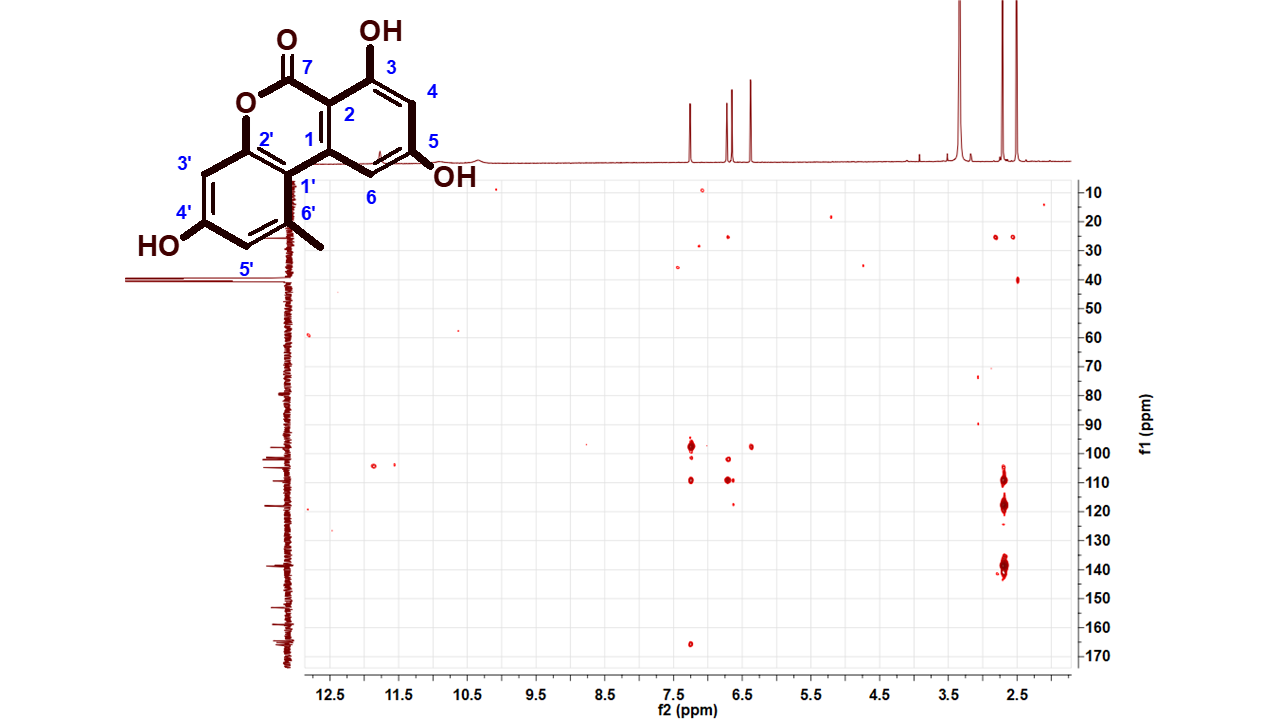
**Figure S13: HMBC spectrum of compound 3.**

**Spectral data of compound 4:**

Compound **4** was isolated as yellow crystals. ESI-MS spectrum of compound **4** illustrated a protonated molecular ion base peak at m/z 167.3 (Figure S14). This information, along with NMR data, suggested C_9_H_10_O_3_ as the molecular formula. ^1^HNMR spectrum of compound **4** showed mulitplets in the aromatic region at δ_H_ 6.04 (2H)–6.09 (1H), which presumed *meta* coupling. Thus, compound **4** was suggested to possess a trisubstituted benzene ring with 3,5-dihydroxyl system. The third substituent was found to have a carbonyl group in addition to two aliphatic singlets as concluded from the ^1^HNMR spectrum (Figure S15).

^13^CNMR spectrum of compound **4** (Figure S16) displayed seven peaks classified as one ketone, two aliphatic carbons and in the aromatic region, two quaternary carbons in addition to two sets of protonated carbons, thus confirming the existence of a trisubstituted benzene ring. Methylene protons detected at δ_H_ 3.45 exhibited HMBC correlations with three carbon resonances at δ_C_ 206.6 (C-8), δ_C_ 136.9 (C-1) and δ_C_ 108.2 (C-2), indicating the presence of a methylene group between the acetyl group and the benzene ring. Additionally, the presence of an acetyl group was confirmed through the HMBC correlation observed between the methyl singlet at δ_H_ 2.07 and the carbonyl carbon at δc 206.6.

Based on the discussed data and by comparison with published literature [5][6], compound **4** was assigned as *α*-acetylorcinol.

**
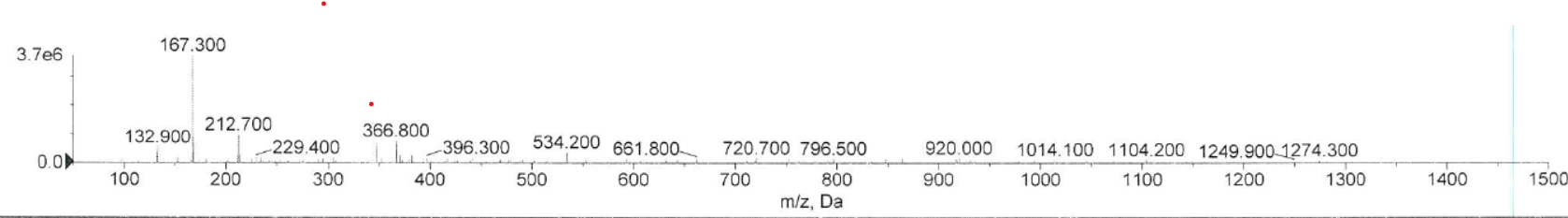
**

**Figure S14: ESI-MS spectrum of compound 4.**


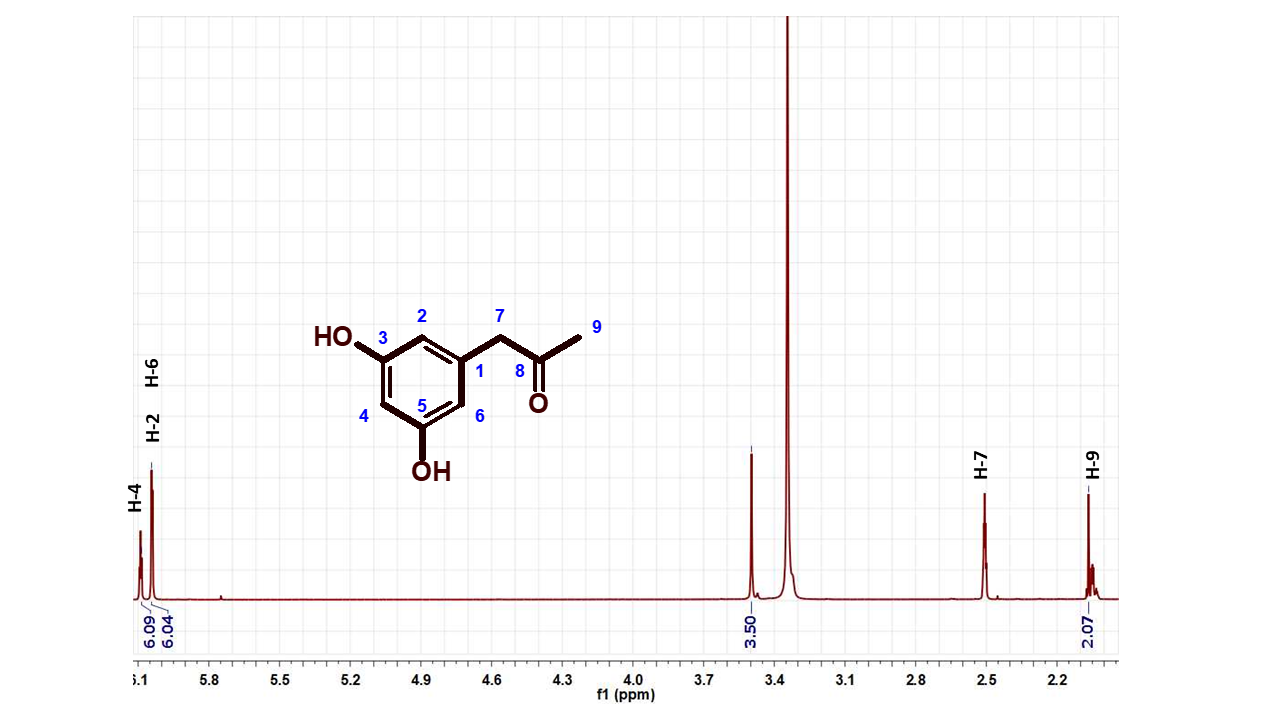
**Figure S15: ^1^HNMR spectrum of compound 4.**


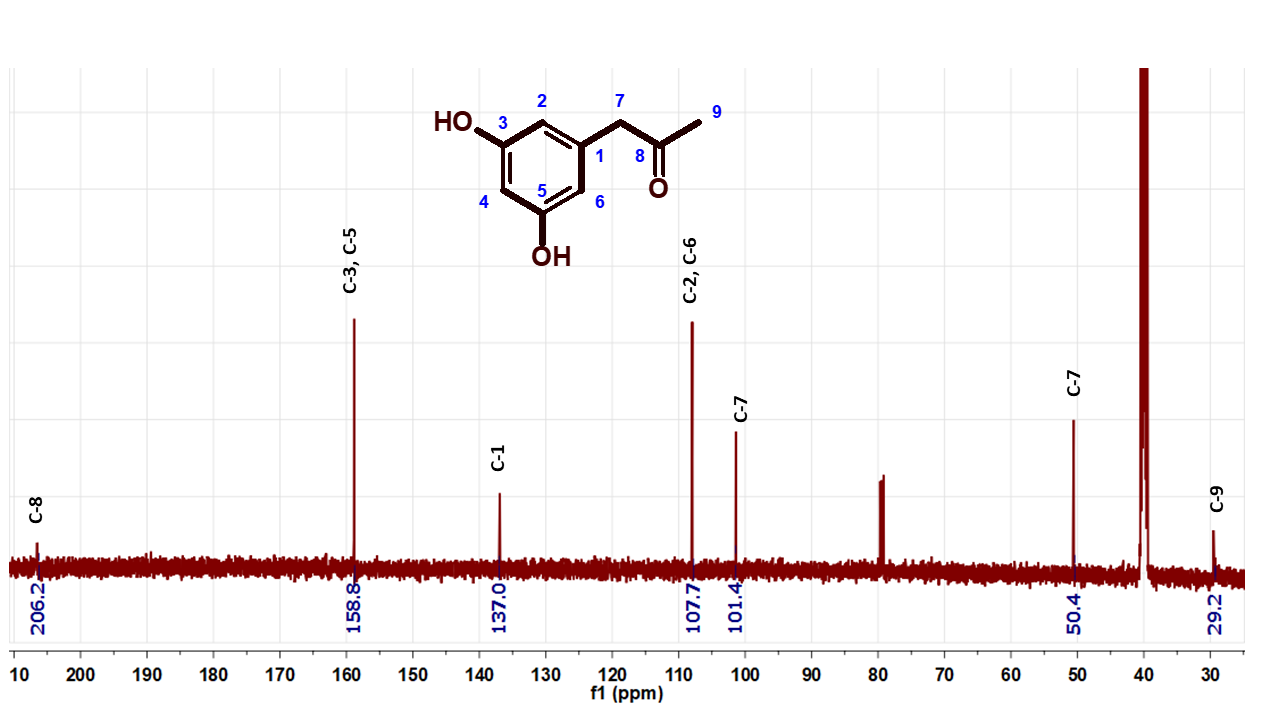
**Figure S16: ^13^CNMR spectrum of compound 4.**


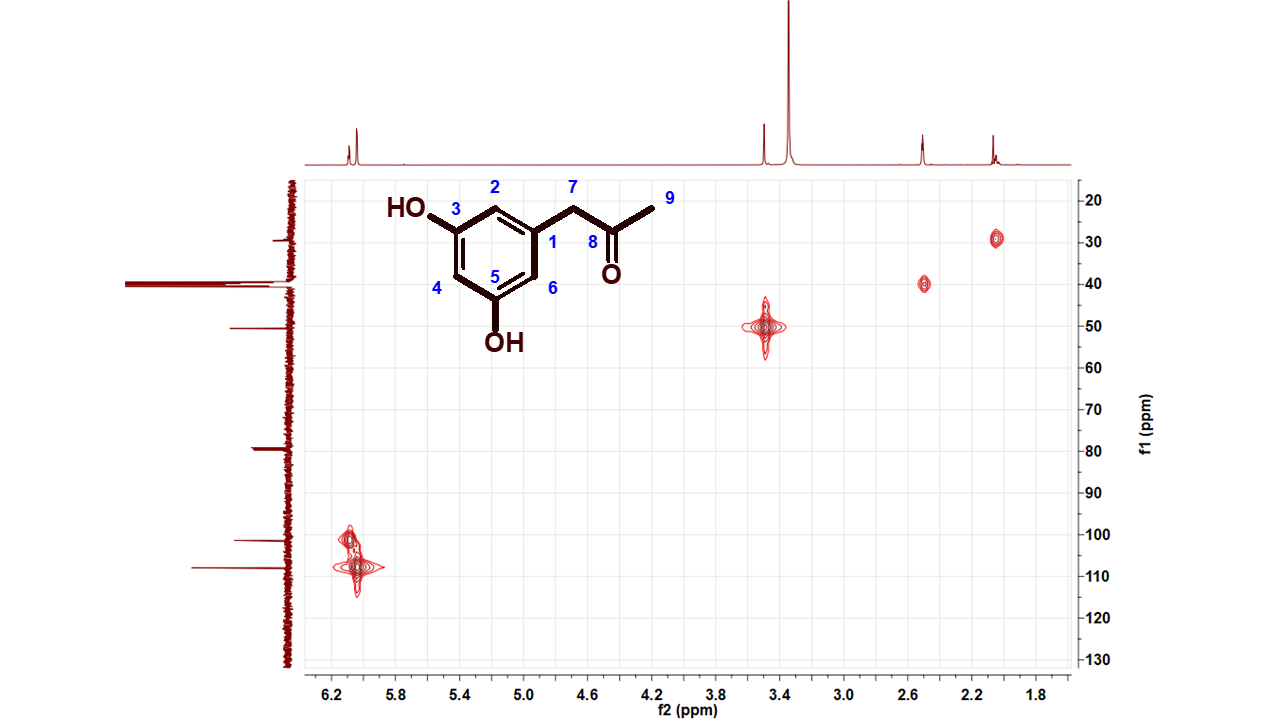
**Figure S17: HMQC spectrum of compound 4.**


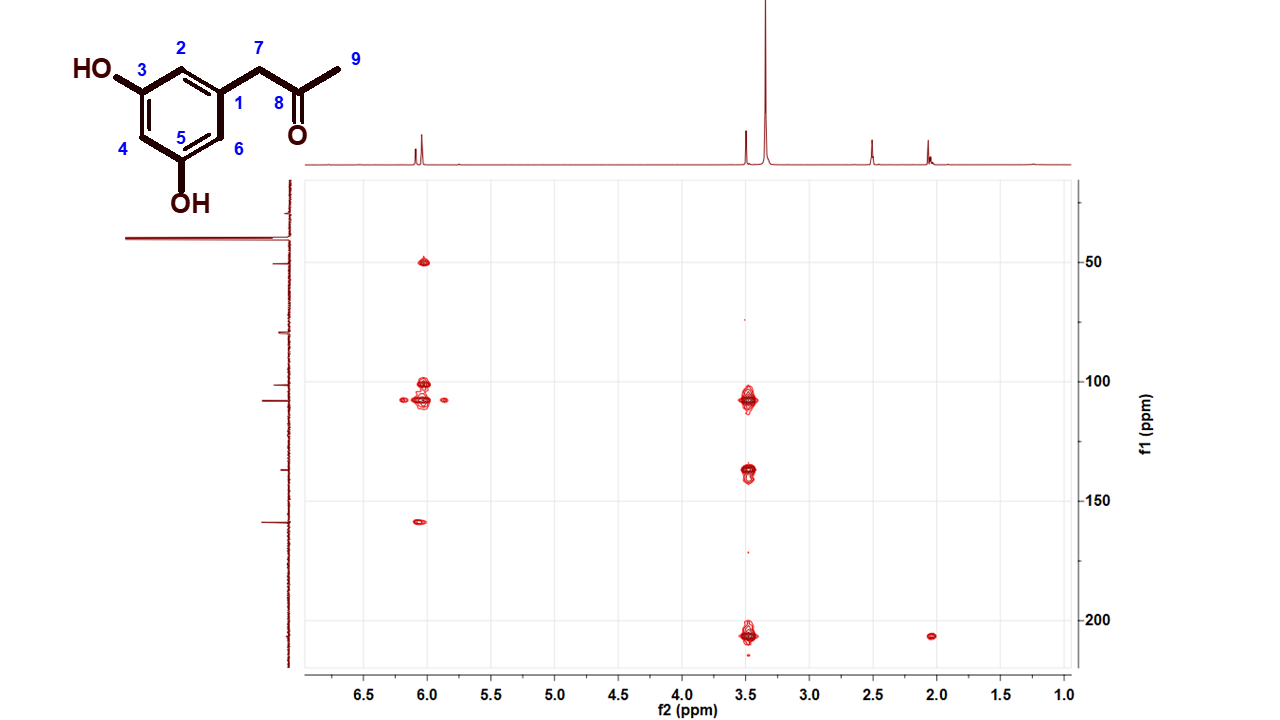
**Figure S18: HMBC spectrum of compound 4.**

**Spectral data of compounds 4 and 5:**

Compounds **5** and **6** were obtained as an inseparable mixture forming a viscous, yellow oil. Their ESI/MS showed a deprotonated ion peak at *m*/*z* 196.1. The characteristic signals for both isomers were identified. The ^1^HNMR data showed the characteristic signals for both isomers (δ_H_ 3.78 (s, H-5), 3.61 (s, H-5), 2.35 (s, H-7), 0.82 (m, H-10), 0.81 (m, H-10)). By comparison with literature data [7], the isomers were identified as tenuazonic acid (**5**) and allo-tenuazonic acid (**6**).

**
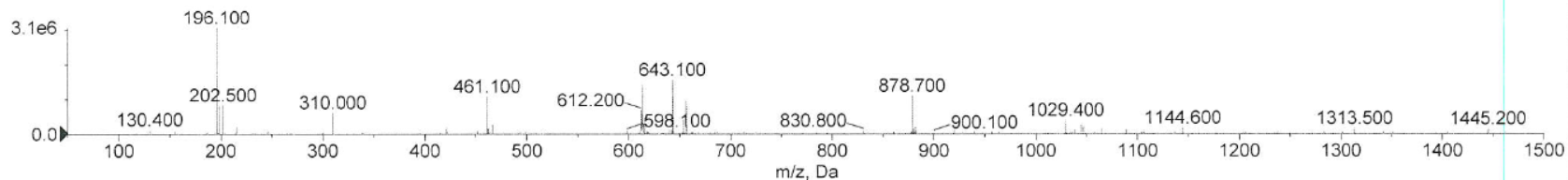
**

**Figure S19: ESI-MS spectrum of compounds 5 and 6.**


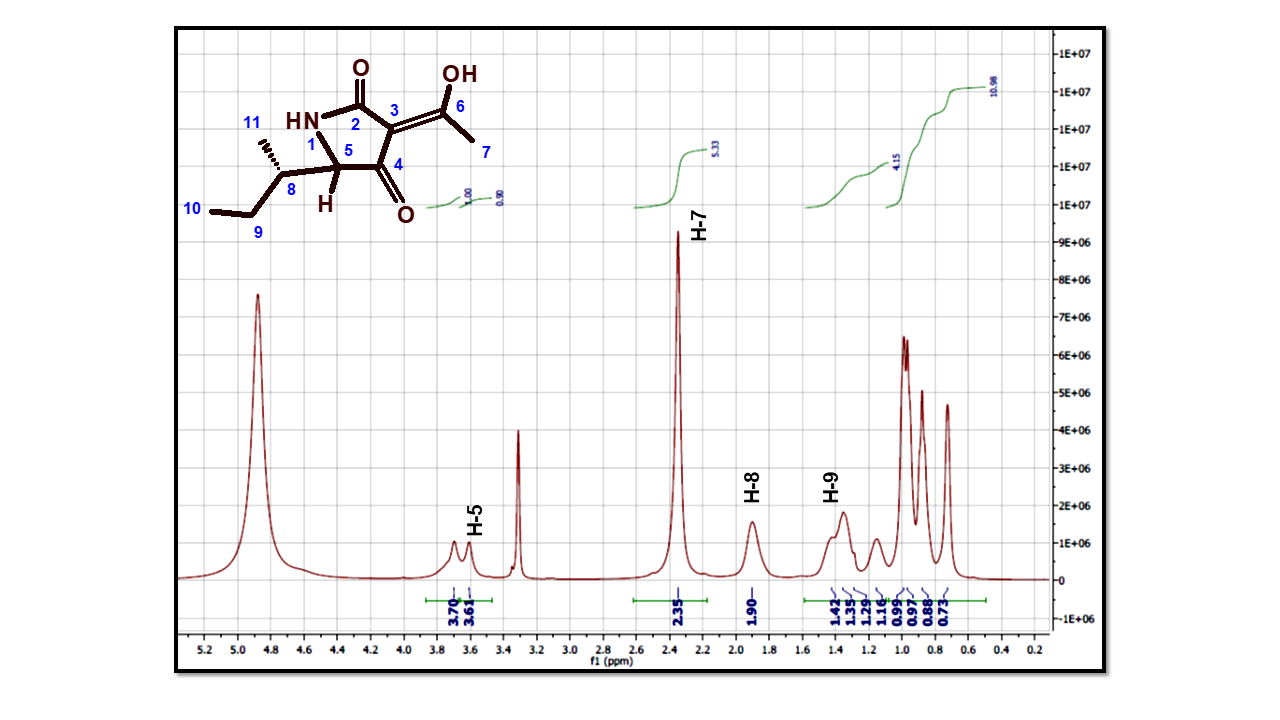


**Figure S20: ^1^HNMR spectrum of compounds 5 and 6.**


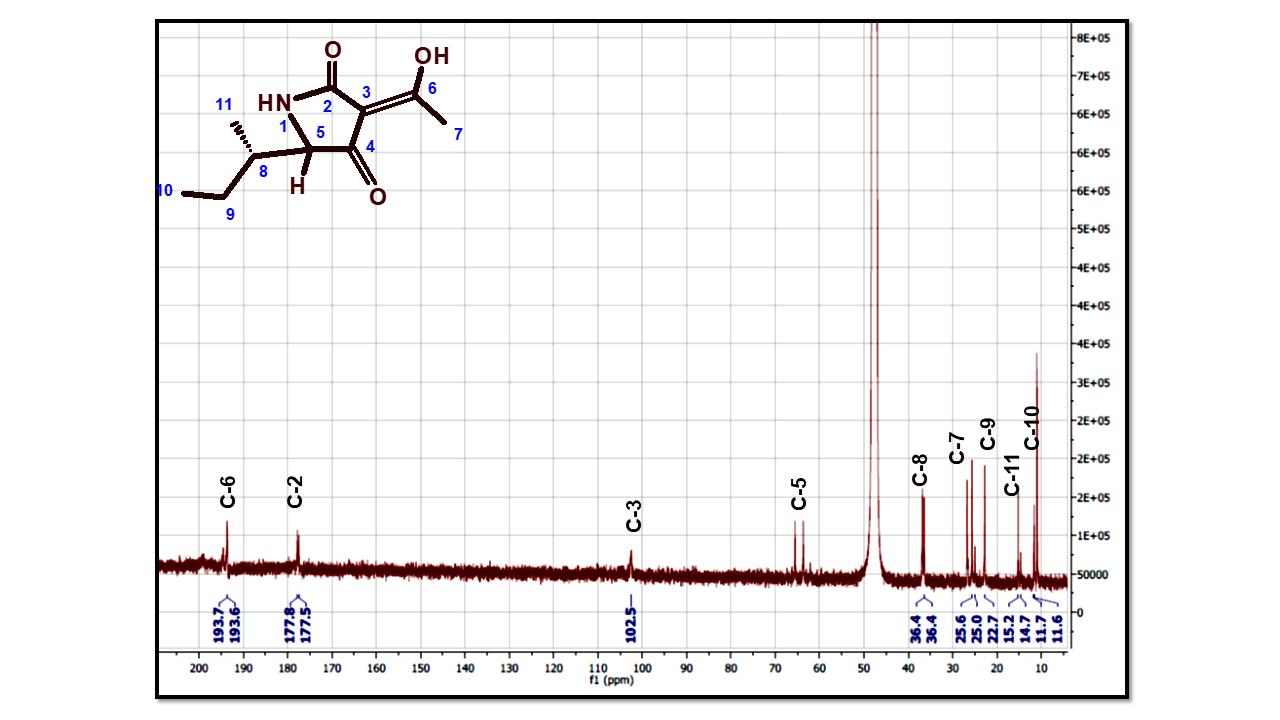


**Figure S21: ^13^CNMR spectrum of compounds 5 and 6.**


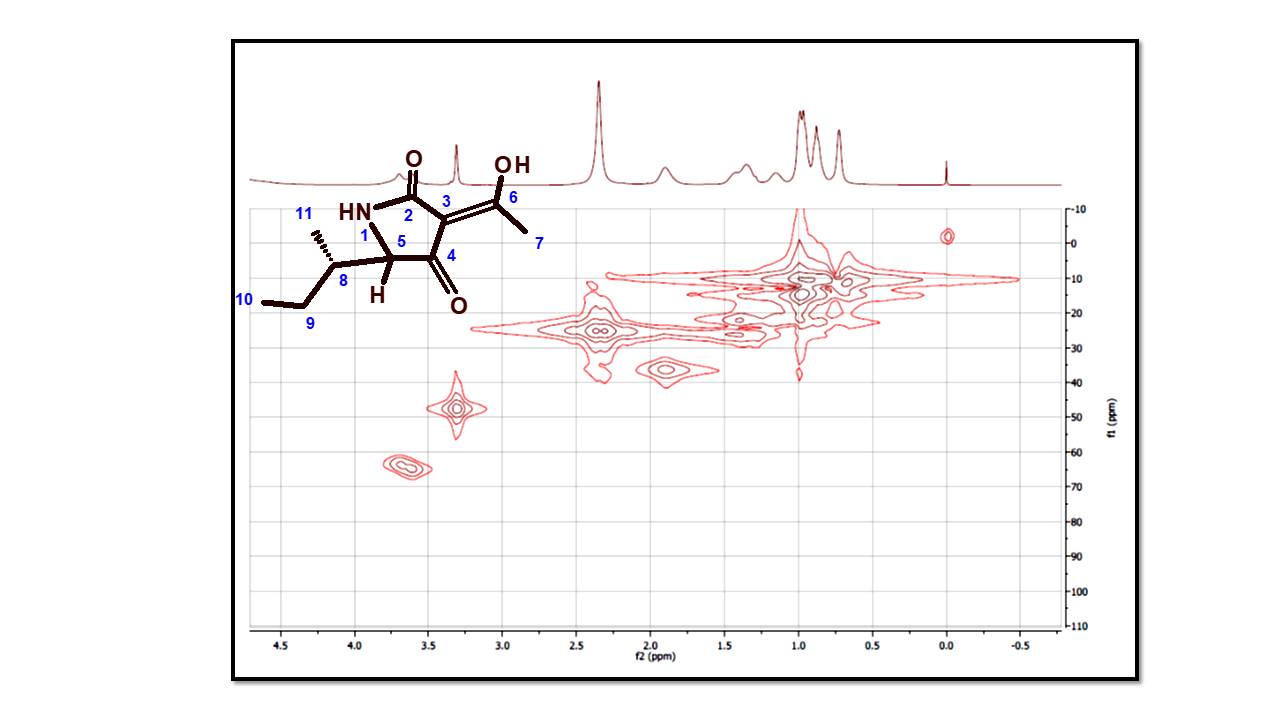


**Figure S22: HMQC spectrum of compounds 5 and 6.**


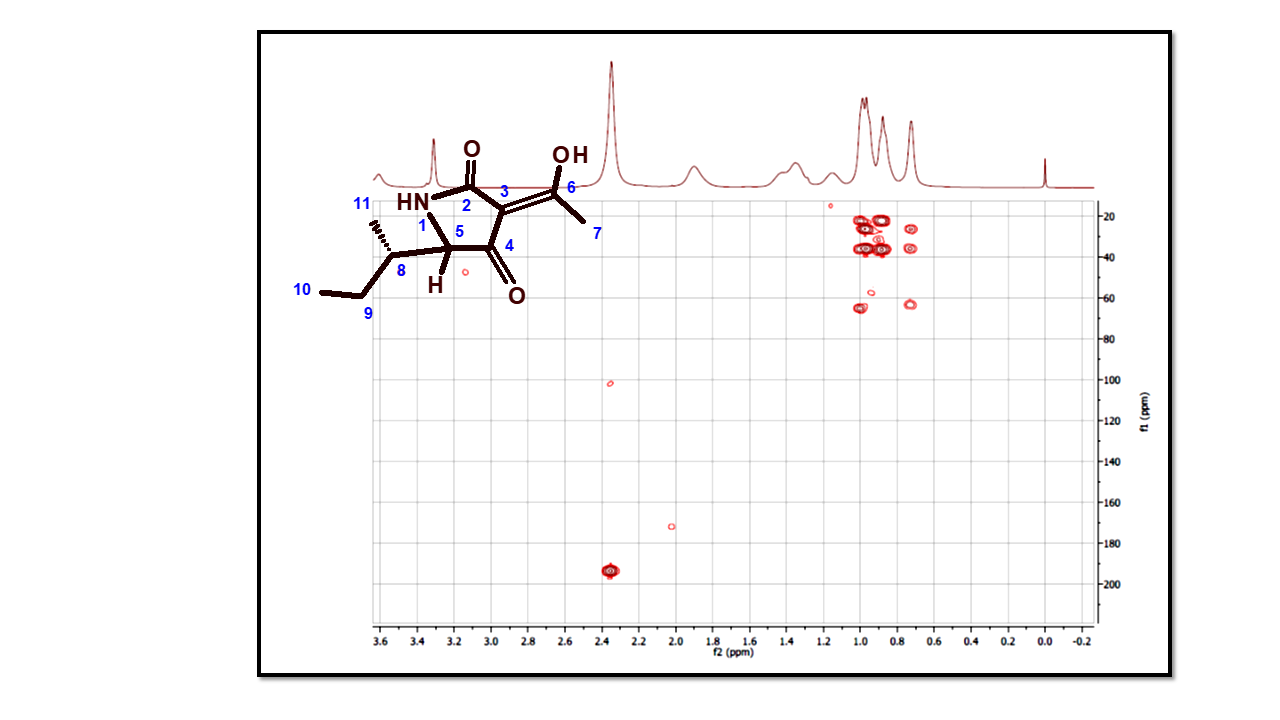


**Figure S23: HMBC spectrum of compounds 5 and 6.**


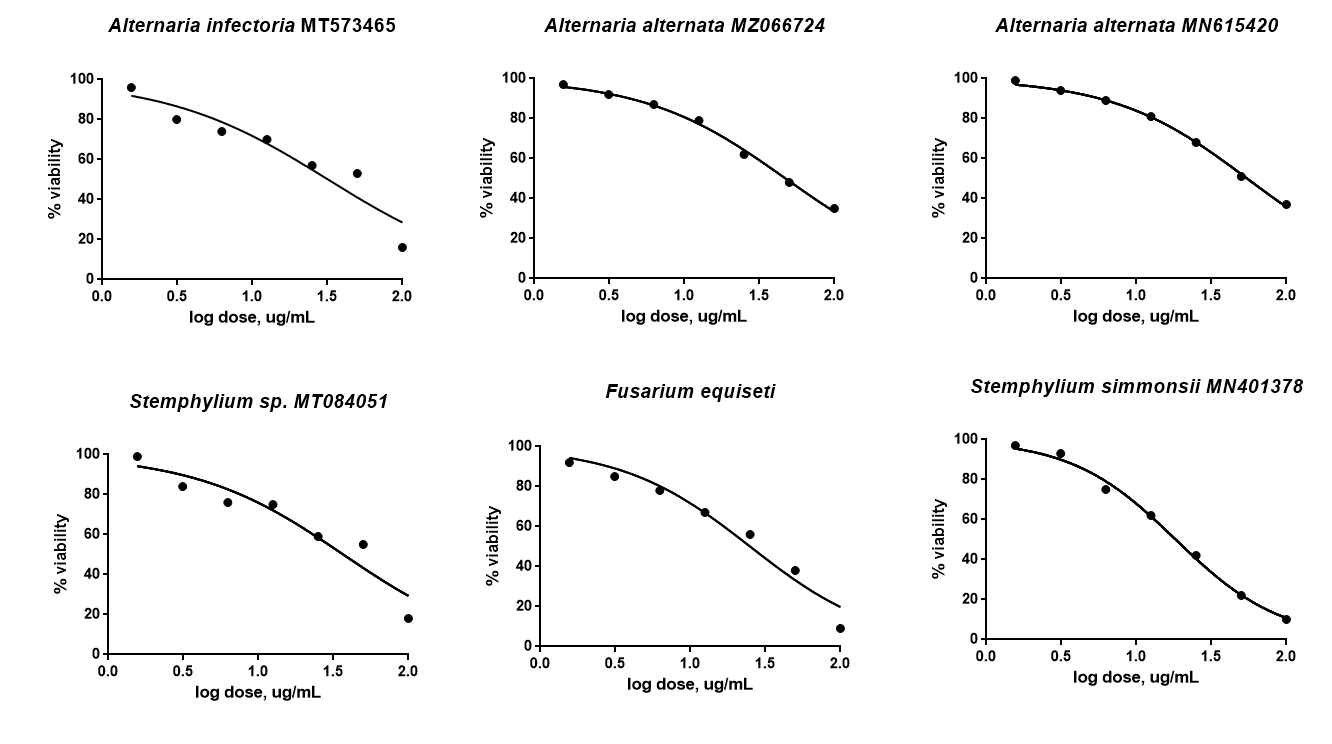


**Figure S24: Cell viability of WI38 exposed to different concentrations of the identified endophytes estimated by MTT assay.(A-K) represent**


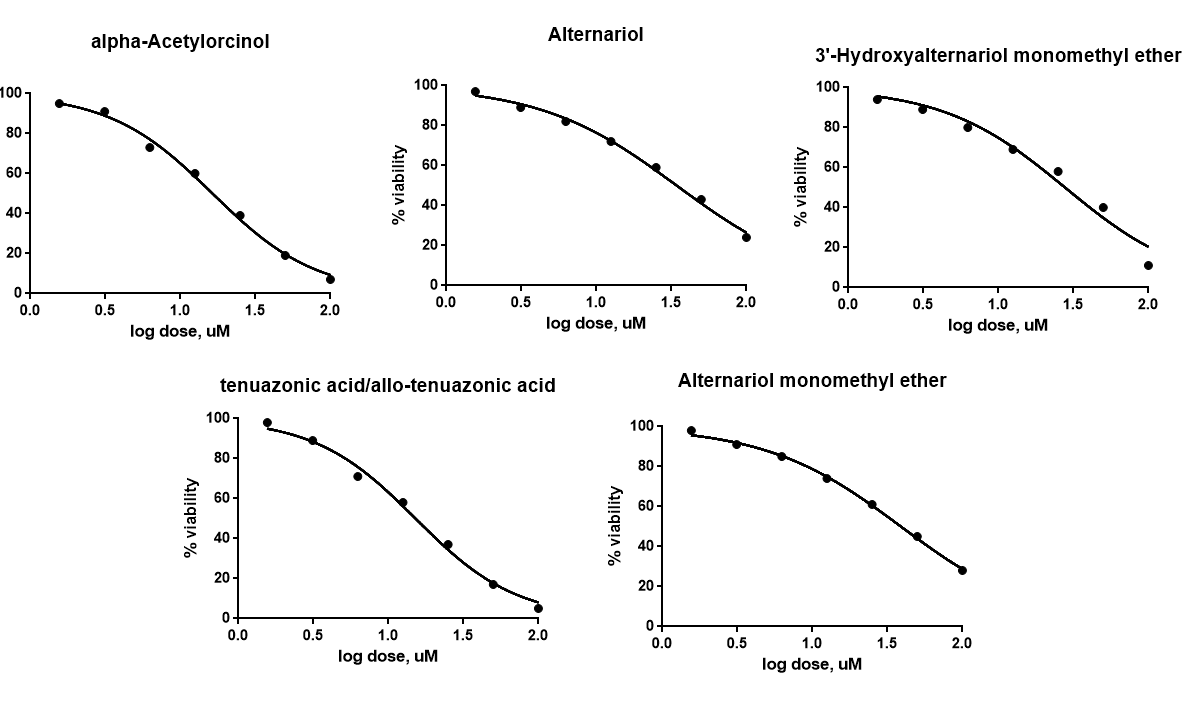


**Figure S25: Cell viability of WI38 exposed to different concentrations of the isolated compounds estimated by MTT assay.(A-K) represent**

**References**

[1] G. D. de Souza, A. Mithöfer, C. Daolio, B. Schneider, and E. Rodrigues-Filho, “Identification of Alternaria alternata mycotoxins by LC-SPE-NMR and their cytotoxic effects to soybean (Glycine max) cell suspension culture.,” *Molecules*, vol. 18, no. 3, pp. 2528–2538, Feb. 2013, doi: 10.3390/molecules18032528.

[2] X. Meng *et al.*, “Benzopyranones from the endophytic fungus Hyalodendriella sp. Ponipodef12 and their bioactivities.,” *Molecules*, vol. 17, no. 10, pp. 11303–11314, Sep. 2012, doi: 10.3390/molecules171011303.

[3] M. Masiello *et al.*, “Molecular Identification and Mycotoxin Production by Alternaria Species Occurring on Durum Wheat, Showing Black Point Symptoms.,” *Toxins (Basel).*, vol. 12, no. 4, Apr. 2020, doi: 10.3390/toxins12040275.

[4] A. Bianchini and L. B. Bullerman, “MYCOTOXINS | Classification,” C. A. Batt and M. L. B. T.-E. of F. M. (Second E. Tortorello, Eds. Oxford: Academic Press, 2014, pp. 854–861. doi: https://doi.org/10.1016/B978-0-12-384730-0.00230-5.

[5] M. S. Pedras and M. R. Park, “Metabolite diversity in the plant pathogen Alternaria brassicicola: factors affecting production of brassicicolin A, depudecin, phomapyrone A and other metabolites,” *Mycologia*, vol. 107, Aug. 2015, doi: 10.3852/15-054.

[6] M. Leyte-Lugo, P. Richomme, P. Poupard, and L. M. Peña-Rodriguez, “Identification and Quantification of a Phytotoxic Metabolite from Alternaria dauci.,” *Molecules*, vol. 25, no. 17, Sep. 2020, doi: 10.3390/molecules25174003.

[7] S. Hickert, I. Krug, B. Cramer, and H.-U. Humpf, “Detection and Quantitative Analysis of the Non-cytotoxic allo-Tenuazonic Acid in Tomato Products by Stable Isotope Dilution HPLC-MS/MS,” *J. Agric. Food Chem.*, vol. 63, no. 50, pp. 10879–10884, Dec. 2015, doi: 10.1021/acs.jafc.5b04812.
